# Experimental Studies of Front-of-Package Nutrient Warning Labels on Sugar-Sweetened Beverages and Ultra-Processed Foods: A Scoping Review

**DOI:** 10.3390/nu12020569

**Published:** 2020-02-22

**Authors:** Lindsey Smith Taillie, Marissa G. Hall, Barry M. Popkin, Shu Wen Ng, Nandita Murukutla

**Affiliations:** 1 Department of Nutrition, University of North Carolina at Chapel Hill, 135 Dauer Dr, Chapel Hill, NC 27599, USA; popkin@unc.edu (B.M.P.); shuwen@unc.edu (S.W.N.); 2 Department of Health Behavior, University of North Carolina at Chapel Hill, 135 Dauer Dr, Chapel Hill, NC 27599, USA; mghall@unc.edu; 3 Vital Strategies, 100 Broadway 4th floor, New York, NY 10005, USA; NMurukutla@vitalstrategies.org

**Keywords:** food labeling, front-of-package labels, obesity prevention, food policy, warning labels

## Abstract

Policies that require front-of-package (FoP) nutrient warnings are becoming increasingly common across the globe as a strategy to discourage excess consumption of sugary drinks and ultra-processed food. However, a better understanding of the pathway through which FoP nutrient warnings work, as well as a review of how outcomes being measured in recent studies map onto this pathway, are needed in order to inform policy on the most effective FoP label design for reducing purchases of ultra-processed foods. This scoping review describes a conceptual model for how FoP nutrient warnings affect consumer behavior, examines which of these outcomes are currently being measured, and summarizes evidence from randomized controlled experiments. Twenty-two studies which experimentally tested nutrient warnings against a control label or other labeling systems were included for full-text review. Our conceptual model includes attention; comprehension, cognitive elaboration, and message acceptance; negative affect and risk perception; behavioral intentions, and behavioral response, along with other elements such as external factors and interpersonal communications. We found that many studies focused on outcomes such as attention, comprehension, and behavioral intentions, but considerable gaps in the evidence remain, particularly for intermediary steps on the pathway to behavioral change, such as negative affect and social interactions. FoP nutrient warnings were visually attended to by consumers, easy to understand, helped consumers identify products high in nutrients of concern, and discouraged them from purchasing these products, although other labeling systems were perceived as containing more information and performed better at helping consumers rank the healthfulness of products. More research is needed to understand whether and how nutrient warnings work in the real world to discourage consumer purchases of sugary drinks and ultra-processed food.

## 1. Introduction

The rapid increase in intake of ultra-processed foods across the globe [1], including in low-and-middle-income countries, poses a major threat to public health. Ultra-processed foods are those made from processed substances extracted or refined from whole foods; most are shelf-stable, ready-to-eat, high in energy density, high in other nutrients of concern (e.g., free sugar, sodium), and low in beneficial nutrients (e.g., fiber) [2]. Large cohort studies have found that diets high in ultra-processed foods are associated with increased risk of hypertension [3], cardiovascular disease [4], overweight/obesity [5], and cancers [6], as well as increased mortality [6,7,8,9,10,11]. Numerous cohort studies have also found that increased intake of ultra-processed foods adversely impacts adult or child health significantly [1,3,4,5,6,7,8,9,12,13,14,15,16,17,18,19,20]. In addition, a recent randomized controlled trial feeding study found that a diet comprised of ultra-processed foods led to an additional 500 kcal/day energy intake and 0.9 kg of weight gain in only two weeks [21]. As a result, scholars, advocates, and policymakers are increasingly calling for policies to discourage consumption of ultra-processed foods and beverages [22].

In the last decade, fiscal policies such as taxes have been one of the most prevalent public policy approaches for reducing intake of sugar-sweetened beverages (SSBs) and ultra-processed foods, with a growing body of real-world evaluation studies showing that these policies reduce purchases and intake of these products [23,24,25,26]. More recently, health scholars, advocates, and international agencies have increasingly called for additional policies that require front-of-package (FoP) warnings on SSBs and ultra-processed foods, in recognition that a package of policy actions is needed to improve diets and prevent further increases in obesity [27]. 

Chile was the first country to implement a mandatory national FoP nutrient warning label policy in 2016 [28], followed by Peru, Uruguay, and Israel [29]. Mexico has approved a similar nutrient warning label law, and number of additional countries have proposed or anticipate federal legislation to require nutrient warnings, including Colombia, Brazil, and South Africa, among others. These warnings typically include text statements denoting high or excess levels of nutrients of concern (frequently referred to as “critical nutrients”), including added sugar, sodium, saturated fat, and in some cases, trans fat, energy or non-caloric sweeteners. The warnings also often, but not always, use shapes, text, or colors intended to signal a warning and to discourage consumption (i.e., a red stop sign or text that says, “avoid excess consumption”).

Evidence on the effectiveness of these “high content” FoP nutrient warnings is needed to inform ongoing advocacy and regulatory processes. Although a number of recent systematic reviews on food labeling have been published, these either do not include nutrient warning studies [30,31] or have grouped together heterogeneous types of labeling on packages, including back-of-package nutrition information as well as positive logos, nutrition claims, and other messages [32]. This limitation may be in part because most studies on nutrient warnings were published within the past several years. However, more fundamentally, the range and heterogeneity of labeling schemes under consideration suggest that core questions about the use and effect of FoP labels remain unanswered. 

In particular, the literature has not yet addressed how the inherent conceptual differences in FoP nutrient warning labels compared to other FoP labeling types may have different effects on consumer behaviors and ultimately diet-related health outcomes. For example, some FoP labeling systems, such as Nutri-score or Australia’s Health Star Ratings system, create summary indices of multiple nutrients, including nutrients of concern as well as beneficial nutrients or ingredients, to present a product’s overall nutritional profile on a continuum from least to most “healthy.” In these labeling schemes, the labeling system essentially does the work of evaluating the overall nutritional profile for the consumer, but the levels at which nutrients of concern are present in the product are not always immediately evident. Other FoP label schemes, like the traffic light label, which color-code multiple nutrients, convey complex and sometimes contradictory information (e.g., a product is high in one nutrient of concern but low in another), requiring consumers to evaluate all the information to come to an assessment of overall healthfulness. This could be particularly challenging for products that are often misperceived as healthy, in categories like yogurt or breakfast cereal, where a product may have red (high) values of one nutrient of concern but green (low) values of another nutrient of concern. In contrast, nutrient warnings are binary, focused on nutrients of concern, and signal to consumers the presence or absence of high levels of these nutrients of concern. These distinctions may have important implications for how labeling systems influence consumer behavior. For example, FoP labeling systems which either do not call attention to nutrients of concern, or potentially present conflicting information, may be more likely to encourage consumers to choose the “healthier” option, potentially among an array of relatively unhealthy products. In contrast, because FoP nutrient warning labels help consumers more rapidly identify unhealthy products through the signaling of the presence of high levels of nutrients of concern, they may be better suited to helping discourage consumers from excess consumption of these products. 

However, to date, the literature on FoP labels has not articulated these key distinctions between labeling systems. A better understanding of the pathway through which the FoP labeling systems work, and the psychological importance of the “warning” aspect of labels is needed, as well as a review of how current outcomes being measured map onto this pathway, in order to inform ongoing research regarding the most effective design for reducing consumer purchases of ultra-processed foods. Thus, the objectives of this scoping review are to: describe a conceptual model for how nutrient warning FoP labels affect consumer behavior; examine which of these outcomes are currently being measured in the literature; and to review the existing evidence on FoP nutrient warnings from randomized controlled experiments with regards to these outcomes. 

### Conceptual Model

Labels on packages are a means of communication to guide consumers’ purchase decisions. While marketers use them to increase the sales of their products [33], they are also used by governments to communicate the risks of products like tobacco and discourage consumption [34]. 

**Figure 1:**
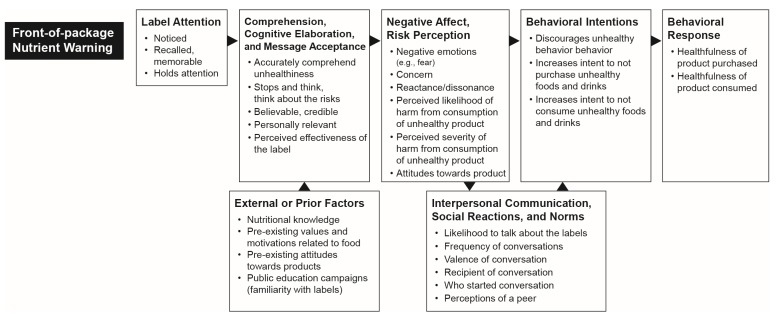
Conceptual model.

The persuasive ability of FoP labels may be explained by principles from social and behavioral sciences, and in particular, theories of persuasive communication. Our conceptual model (Figure 1) draws on decades of these theories [35,36,37,38,39,40], and builds on models developed for tobacco pack warnings [41,42], adding key constructs relevant to the nutrition context. In summary, our model suggests that for warning labels to be effective, they must first grab attention and be accurately understood. Thereafter, labels must elicit a negative affect or perception of risk, which in turn is expected to trigger behavioral intentions, and ultimately behavior change. External factors may moderate the label’s acceptance and perceived effectiveness. In particular, preexisting values and attitudes towards food, associations with the label from previous exposures, and current nutritional knowledge are among the prior or external factors that influence interpretation and acceptance of a label’s message. Finally, the model suggests that the interpersonal communication triggered by labels also plays an important role in reinforcing desirable behaviors, such as the avoidance of unhealthy foods.

More specifically, fundamental to a FoP nutrient warning’s effectiveness is that it catches the consumer’s attention and is accurately understood. People often make snap decisions that are not based on “rational” or deep processing of information, and this is particularly so when they are less engaged or personally invested in a situation [37]. Consumers make decisions very quickly (in seconds) [43], and food marketers exploit this by using eye-catching design features and product claims to attract the sale of products [44]. In this context, FoP nutrient warnings must not only cut through the other design elements and catch a consumer’s attention, but also provide information that is quickly but accurately understood and signals relevance to the consumer’s subsequent decisions [45]. Secondly, FoP nutrient warnings must motivate the consumer’s product choice. Yet, as shown in a number of public health areas—from tobacco use to road safety behaviors—knowledge of a health risk is not sufficient to motivate people to desired actions. According to psychological theory, for behavior change to occur, the risk must be perceived as likely and severe [46,47], and people must see themselves as personally susceptible to it. Indeed, communication interventions for a number of behavioral risk factors, including tobacco use, have sought to achieve behavior change by highlighting the perception of severe risk and by increasing personal susceptibility [48]. Other psychological theories have described the critical mediating role of negative affect and the fear of personal loss in achieving such behavior change [41,47]. When “low risk” events are reframed as probable losses, they are more likely to motivate action [49,50]. Likewise, the generation of dissonance [51]—or the uncomfortable feeling triggered by the discordance between a belief and a behavior—motivates corrective behavior as a way to reduce the discomfort. Finally, work in the area of behavioral decision-making has found that when people make decisions with uncertain or incomplete information, their choices are systematically guided by a powerful heuristic to avert losses and maximize gains [49,52]. In sum, there is general convergence between a number of psychological theories of the important role played by negative affect and the motivations to minimize risk and avert losses in behavior change. In fact, these theories have been tested extensively for tobacco graphic health warnings, and the negative health consequences of tobacco use were found to be the most effective in motivating tobacco users to reduce their consumption [41,42]. In the context of discouraging the consumption of ultra-processed products, FoP nutrient warnings may be expected to play a similar role in countering the immediate gratification of these products by reminding consumers of the increased health risks and potential loss of good health from their excessive consumption. Thus, once the FoP nutrient warning motivates consumers, it is expected to lead to increased behavioral intentions following by increased behaviors to reduce consumption of unhealthy products.

Finally, the effectiveness of FoP warning labels may be affected by a complex array of external factors, including preexisting information, attitudes, motivations, and values, community norms, and environmental cues [53]. For example, preexisting attitudes towards a particular food, associations with the label from previous exposures, and current nutritional knowledge may affect the label’s acceptance and perceived effectiveness. In addition, substantial literature has found that social norms—often measured by interpersonal communication and perceived social approval—exert a powerful influence on behavioral intentions [54,55]. Thus, labels that trigger conversation and that signal social disapproval are likely to be more effective.

## 2. Materials and Methods

The scoping review was conducted according to the guidelines established by PRISMA (Preferred Reporting items for Systematic Reviews and Meta-Analyses) (see PRISMA checklist, Table S1).

### 2.1. Search Strategy

The databases Google Scholar, PubMed, Medline, Psych Info, and Scopus were searched for articles published in English-language journals between 1 January 2014 and 1 September 2019. The last search was conducted on 2 October 2019. Reference lists from eligible studies and systematic reviews were also searched for additional relevant studies. Peer-reviewed studies were included; grey literature and self-published studies were excluded, as were non-English-language studies. 

The search terms aimed to identify randomized experiments on nutrient warnings for foods or beverages (Table S2) and included “warning” or “label;” “pack,” “package,” or “front-of-package;” “food“, “drink“, “beverage,” or “snack;” and “random,” “randomized,” “trial,” or “experiment.” 

Studies eligible for inclusion were those that examined the impact of nutrient-based front-of-pack warning labels on food or beverage packages on outcomes relating to constructs in our conceptual model (i.e., attention, comprehension, message acceptance, negative affect/risk perception, behavioral intentions, interpersonal communication, or behavioral response). Studies that employed a randomized design (within or between subjects) were included, thereby excluding natural experiments, observational studies, or pre-post evaluations. We included only randomized experiments because they are the gold standard for demonstrating the causal impact of new interventions [56], including warning labels. 

**Figure 2:**
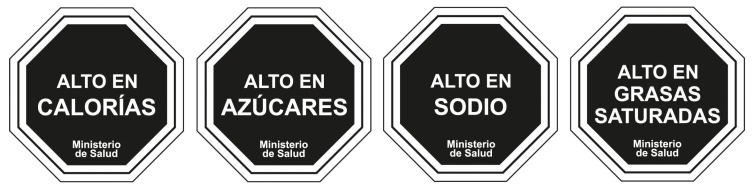
Example of a front-of-package (FoP) nutrient warning label system from Chile. In English, the labels say, “high in calories,” “high in sugars,” “high in sodium,” and “high in saturated fats,” respectively, with “Ministry of Health” noted at the bottom.

A nutrient warning was defined as a label that conveys information that a product contains high or excess levels of specific nutrients (sugar, saturated fat, sodium, or energy) or any amount of other nutrients of concern (trans fat or non-caloric sweetener) (Figure 2). Studies that examined only other types of FoP labels, such as health warnings (images/and or text statements linking consumption of a product to a health outcome), Guideline Daily Amounts (GDAs), positive logos, traffic light labels, Nutri-score, or the Health Stars Rating System were not included. We included only studies that focused on labels on the front of food and beverage packages; studies that focused only on labels on menus, store shelves, vending machines, advertisements, or in cafeterias were excluded. We included studies with any or no control (i.e., eligible studies compared nutrient-based warnings to other nutrient warnings, a no warning control, other FoP labeling systems, or other controls).

Studies that did not use a randomized experimental design to examine the impact of an FoP nutrient warning on an outcome were excluded, such as studies using qualitative methods only (e.g., focus groups), Williams Latin Square method, conjoint-choice analysis (e.g., all participants view the same choice sets comprised of two warning labels with different designs [57]), or studies where effects of the nutrient warning were not tested separately from other experimental manipulations (e.g., randomized to see warnings with or without a claim [58]). 

### 2.2. Study Selection

Two investigators independently conducted title and abstract screening, with any conflicts resolved by consensus. Investigators screened study titles and abstracts to identify potentially relevant articles. Investigators then screened full-text articles against the eligibility criteria, with reasons for exclusion documented. Finally, investigators reviewed references in each included article and screened these against the eligibility criteria.

### 2.3. Data Extraction

For each study, a single coder extracted data on the country, setting (online, laboratory (defined as any in-person, artificial setting), school, or store) and sample demographics, including sample size, age group, percent female, and education (educational level for adults, and type of school (public or private) for children). We also extracted data on study design, which label types were tested, and which outcomes were measured, and whether modification by education level was measured, and summarized the results qualitatively. 

## 3. Results

**Figure 3:**
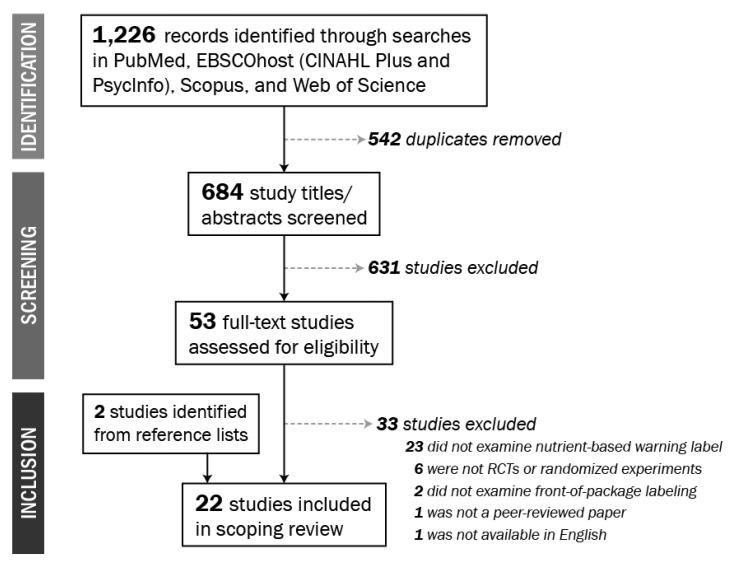
Study selection.

Of 1226 articles identified in our search, 22 studies were included in this review (Figure 3).

A description of study characteristics is shown in Table 1. More than half of the studies took place in Latin America, followed by the US/Canada and Europe/Australia/New Zealand. The majority of studies were conducted online (64%) and among adults (91%). With regards to education, most studies (68%) reported educational attainment, while 18% of studies (all of which included children or adolescents) reported school type. Few studies examined differences in outcomes by education (14%). 

With regards to the type of nutrient warnings tested, octagons were the most common (77% of studies), followed by triangles (18%), and circles (14%). Sugar was the most common nutrient included (91% of studies), followed by sodium (59%) and saturated fat (55%), while only 9% of studies examined other nutrients (e.g., trans fat or non-caloric sweeteners). With regards to outcomes, comprehension was the most common category of outcomes tested (50%), followed by behavioral intentions (41%) and cognitive elaboration and message acceptance (36%). Attention and behavioral response were each tested in 23% of the studies, while negative affect and risk perceptions were tested in 18%, and other outcomes were tested in 14% of the studies. 

More detailed information about each study including the design, stimuli, outcomes, and summary of evidence by outcome can be found in Table 2 and Table 3. The nutrient warnings used in each study are in Table S3. The comparison FoP labels tested in each study are in Table S4. Full information about the design of each study can be found in Table S5.

Attention: With regards to attention, nutrient warnings were visible, and participants paid attention to them [66,73]. In one study that compared nutrient warnings to traffic light labels, participants rated the nutrient warnings as having higher visibility and drawing more attention [66], though in a separate study, nutrient warnings were rated similarly as other labels in terms of whether they stood out [78]. An eye-tracking study found that half of participants fixated on nutrient warnings when viewing food packages [76].

Comprehension: With regards to comprehension, results were mixed and dependent on the types of labels being tested as well as the measures being used. Compared to a no-label control, nutrient warnings improved consumers’ ability to identify unhealthy products (with excess nutrient content) [65,66,73], in some cases, more than other labeling types, such as traffic light labels [66], and also reduced consumer perceptions of healthfulness [67]. An additional study found that compared to a no-label control, nutrient warnings had a bigger impact on reducing perceptions of healthfulness than did the Health Star Rating or Nutri-score [79]. In a multi-country study where participants rated their perceptions of labels, participants in the nutrient warnings condition rated these labels the lowest on “took too long to understand” (reference intakes were highest) and highest for “easy to understand” (Nutri-score was the lowest) [78]. However, consumers also rated traffic lights the highest for containing the most information needed [78]. In addition, other studies found that nutrient warnings did not affect consumers self-reported nutrition knowledge [61] or knowledge of health harms associated with consuming unhealthy products (e.g., SSBs) [72]. In addition, when participants were asked to rank sets of three products according to healthfulness, all labels improved consumers’ ability to correctly rank products compared to a no-label control, but nutrient warnings did not improve the percent of correct responses as much as other labeling types (e.g., Nutri-score) [64,77,80].

Cognitive elaboration and message acceptance: Within this category, outcomes mainly focused on message acceptance. Nutrient warnings tended to be rated as either similarly useful [61] or favorably perceived [77] compared to other labels. In another study, nutrient warnings were rated as having higher usefulness and credibility than traffic light labels [66]. With regards to different shapes, one study found similar ratings of usefulness across shapes (e.g., triangle vs. octagon) [73], while another study found that participants rated the octagonal stop sign as the most preferred symbol, followed by a triangle with an exclamation point and a magnifying glass rated as least preferred [65]; a third study found that octagons were rated as having higher perceived message effectiveness than rectangles [72]. With regards to harshness, one study found that the majority of respondents thought that nutrient warnings were “about right” or “not harsh enough” [62]. With regards to perceived message effectiveness, nutrient warnings were perceived as more effective than a no-label control, but less effective than a health warning [72]. However, participants did not necessarily like the nutrient warnings: in one cross-country study of label perceptions, participants rated nutrient warnings the lowest and second lowest for liking and trust compared to other labeling types [78].

Negative affect and risk perception: With regards to affect, a study of children found that nutrient warnings had a bigger effect on reducing the use of positive emojis (a measure of children’s emotional associations towards unhealthy foods) during product evaluations than did traffic light labels [75]. Nutrient warnings led to increased thinking about harms and fear, though this was less than health warnings [72]. With regards to consumers’ attitudes towards products, parents viewing nutrient warnings reported lower ideal consumption of products containing the warning than did those viewing products with the guideline daily allowance [67]. Another study found that nutrient-based warnings reduced product preferences [59], though less than graphic warnings [59].

Behavioral intentions: With regards to behavioral intentions, findings were mixed. A number of studies found that nutrient warnings reduced participants’ preference for a product [60] and intended purchases [59,66,73,79] of products high in nutrients of concern compared to other labels or a no-label control. However, other studies found null or mixed results. For example, one found that while participants in both nutrient and health warning conditions intended to purchase a lower proportion of high-in-sugar products, this was only statistically significant for the health warnings condition [71]. An additional study found that nutrient warnings did not influence the share of ultra-processed foods consumers intended to purchase, or the mean amount of sugar, calories, saturated fat, and sodium, but did decrease intended purchases of sweets and desserts [68]. A similar study found that compared to a no-label control, nutrient warnings improved the average healthfulness of consumers’ intended purchases, though this improvement was similar to the traffic light label [69]. Finally, one study found that, compared to a reference intake label, no labels influenced intentions to purchase, with the exception that nutrient warnings led to increased intentions to purchase breakfast cereals [77].

Behavioral response: With regards to actual behavioral outcomes, most studies found that nutrient warnings improved the healthfulness of food purchases. Nutrient warnings reduced participants’ choice of snacks and drinks high in critical nutrients [74,76], the level of critical nutrients in beverages and snacks purchased [70], or improved the overall nutritional profile of purchases [61] compared to a no-label control or to other labeling types. However, in another study of purchases, there was no statistically significant effect of warning labels, but there was a trend for nutrient warning labels to reduce purchases of sugary drinks [63].

Other outcomes: Other outcomes not in our conceptual model included support for labeling policies and self-efficacy. One study, which assessed self-efficacy, found that nutrient warnings increased participants’ sense of control over healthy eating decisions, and this increase was larger than the comparison label (Health Star Rating) [62]. With regards to policy support, one study found that the majority of participants agreed or strongly agreed that sugary drinks should carry a nutrient-based text warning [59]. In another study, participants rated warning labels as similar to or slightly lower than other labeling types such as Health Star Rating or traffic lights as to whether it should be compulsory for the label to be shown on packaged food [78].

Modification by education: Only one study examined whether there was modification by educational attainment, and found that education did not modify the effect of labels [72]. Two studies of children examined modification by school type (public vs. private); one study found that labels had a greater effect on private school children [67], and a second study found that labels tended to have a greater effect on public school children [75].

## 4. Discussion

Our conceptual model for how nutrient warnings change behavior includes: attention; comprehension, cognitive elaboration, and message acceptance; negative affect and risk perception; behavioral intentions and behavioral response, along with other elements such as external factors (e.g., prior preferences or knowledge) and interpersonal communications. In this scoping review, we found that the majority of studies tested the effectiveness of FoP nutrient warnings on only a few key outcomes in this model: attention, comprehension, and behavioral intentions. Other crucial intermediary steps in our conceptual model, such as the ability to increase perceptions of risk or negative affect and the ability to trigger interpersonal conversations about the labels, were less frequently tested. This absence of focus on the intermediary steps, which has been demonstrated as crucial for motivating behavioral change in tobacco pack labeling, suggests an important gap in our understanding of how nutrient warnings work. Additionally, behavioral outcomes, such as selection, purchase, or consumption of a snack or drink, were less frequently tested, perhaps because the majority of the studies took place in an online setting which makes testing behavioral outcomes more difficult. This lack of behavioral outcomes is a major gap in the literature, since changes in food purchases and subsequently changes in food intake are needed in order to achieve health goals such as obesity prevention, which are typically the underlying motivation for implementing FoP labeling policies. 

Our review found that FoP nutrient warnings tended to be perceived as visible, credible, and easy to notice and to understand. From the current set of studies, it is not clear why this may be the case. One possibility is that nutrient warnings require less processing compared to the interpretative labels that may require more deep thinking to fully understand the information. For example, a traffic light label can contain red, yellow, and green colors, signaling both high and low levels of nutrients of concern, requiring the consumer to consider which nutrient(s) to prioritize when making a choice. This is exemplified in the Talati study, in which consumers rated traffic light labels as containing more useful information, but the warning labels as being easier to understand [78].

Additionally, the increased “cut through” or visibility of warnings may be explained by social psychological theories that have suggested that people are generally more attentive to negative information, including threats and the fear of loss [81]. Since nutrient warnings only focus on what not to eat, they may imply a clearer picture of what consumers could lose by eating unhealthy foods compared to other systems that are intended to communicate information about both healthy and unhealthy foods. 

However, one study found that nutrient warnings were perceived as not containing all the information consumers need or want [78], and that consumers may not like nutrient warnings as much as other labels. Several studies also found that nutrient warnings tended to be less effective than other label types at helping consumers rank the order of healthfulness of products [64,77,80]. However, nutrient warnings did help consumers identify the relatively unhealthy products (e.g., those containing high levels of nutrients of concern) [65,66,73] and the relatively healthy products [66,73], and led to lower perceived healthfulness of products [67,79]. This makes sense because nutrient warnings only contain information about high levels of nutrients of concern, and overall, they contain less nutritional information than other systems, such as traffic light labels or Nutri-score, which summarizes both nutrients and ingredients with a color-coded “grade” from A–E. This suggests that nutrient warnings are better for helping consumers making binary distinctions (e.g., identifying that a product is unhealthy), rather than helping them rank products by overall healthfulness. Interestingly, one study found that the speed (or ease) with which consumers are able to evaluate healthfulness depends on whether the product is healthful or unhealthful. Warning labels have a shorter processing time when consumers are evaluating unhealthy products, which suggests that they perform better at helping consumers identify unhealthy products rather than assess the healthfulness of relatively healthy products [79]. 

Thus, while nutrient warnings appear best suited to enable consumers to identify relatively unhealthy products, other labeling systems that provide more information appear to be better for helping consumers rank the healthfulness of products. However, this feature of the more informative labels could be one reason why they could be less effective at changing behavior: they likely require consumers to quickly compute and interpret more complex information compared to nutrient warnings. The ease of interpretation and use of FoP systems is particularly important given that consumers are often making purchasing decisions while distracted by their children (e.g.., pester power) or while experiencing other forms of cognitive load (e.g., determining their spending budget, or responding to visual/audio and other sensory stimuli in the store setting). In other words, the simplicity with which FoP nutrient warnings convey pertinent information may be what makes them effective at reducing unhealthy food purchases: they reduce the information to a set of binary labels, and therefore point consumers to binary decisions (buy or not buy). 

It is also important to note that our conceptual model is focused on consumer-level factors on the pathway to behavioral change. Thus, our model may not capture all the elements required for a labeling system to be effective at improving consumers’ diets in the real world [82,83]. For example, other elements of the labeling regulation such as whether the system is mandatory or voluntary can strongly affect the likelihood of a labeling system to change consumer behavior. One concern with voluntary systems is that the labels will appear only on products that are already somewhat healthy and be omitted from unhealthy products. This has already been seen for some voluntary systems, like Australia’s Health Star Ratings System, with one evaluation finding that products carrying the Health Star Rating are more likely to be healthy than products that do not [84]. The influence of these important regulatory elements may be difficult to include in an experimental setting; indeed, none of the studies included in this review tested this. Instead, most experimental studies assign labels to products in an idealized scenario that may not reflect the real world (e.g., even unhealthy products receive the voluntary Health Star Rating). For this reason, natural experimental work evaluating real-world policies as they are implemented will be needed in order to understand the real-world impact of these labeling systems on behavioral change.

An additional element of food labeling regulations that was not included in our model nor tested in experimental studies relates to the nutritional profile model that underlies a FoP nutrient warning system. In fact, the nutritional profile model used differed across the studies, making it challenging to compare them. For example, sometimes the nutrient thresholds from Chile’s Law of Labeling and Advertising were used; sometimes, the nutritional profile from the Pan American Health Organization (PAHO) was used; and sometimes, another nutrient profile was used. These systems not only apply labels to different nutrients (e.g., Chile’s model includes a calories label, whereas PAHO includes labels for total fats, trans fats, and non-caloric sweeteners), but also use different algorithms or reference values to determine which products receive label(s). These different nutrient profile models will influence what nutrients are included and how many products are covered [85,86,87,88], with potentially major differences in what receives a warning label depending on the food category. In addition, other labeling systems incorporate nutrients of benefit into their summary score calculations, with the underlying assumption that some beneficial nutrients like vitamins, fiber, or fruit and vegetable content offset the negative effects of other critical nutrients, such as sugar or sodium. Yet, there are no extant studies that show that fiber or any vitamin or mineral can offset the negative effects of high levels of sugar, sodium, unhealthy saturated fats, or the presence of trans fats. In addition, the amount of some nutrients or ingredients is not always required to be reported on the label, making assessment of the appropriateness and accuracy of the indices difficult. More research is needed to understand how the different nutritional profile models that underlie FoP labeling systems influence consumers’ ability to use and understand FoP labels and ultimately impact consumers’ choice of what to buy and eat.

Future studies might also examine potential external or prior factors at the consumer level, such as levels of nutritional knowledge or familiarity with the labels as potential modifying factors that would enhance or deter a label’s effectiveness. Similarly, no study looked at how mass media campaigns influence the impact of nutrient warnings, which is important, since tobacco control studies found that mass media campaigns paired with pictorial warnings have multiplicative or additive effects [89,90]. Studies that consider these external or prior factors will be important for understanding how nutrient warnings may operate in real-world settings.

Finally, very few studies examined differences in label impact by education or other education-related factors that may be relevant, such as literacy. One study found that education did not modify the effect of labels [72]. Two other studies, focused on children, used public vs. private school as the way of differentiating education, though this measure may be a broader measure of socio-economic status, reflecting income of the parents as much or more than the quality of the education. In these studies, results were mixed, with one study finding that labels influenced only private school children [67], while a second study found that public school children were more responsive to labels [75]. More research is needed to understand whether there is a differential effect of nutrient warnings by education as well as other socio-economic factors, such as literacy, which could influence consumers’ ability to comprehend the labels, and income level, which could influence consumers’ ability to shift between products. 

This scoping review has several limitations. The search criteria we used meant that studies that did not include the words randomized, trial, or experiment in their abstract or title were excluded; therefore, it is possible that we missed eligible studies that may not have included these terms in their abstract or title. In addition, because this is a scoping review, we did not perform a quality assessment of studies. In addition, there was considerable heterogeneity across studies, preventing us from quantitatively synthesizing the results as the literature on this topic continues to expand. Future studies should conduct meta-analyses of results across a more similar set of experiments. Finally, we only included results about the main effects of nutrient warnings compared to a no-label control or other labeling systems. A growing number of studies are testing the interaction of nutrient warnings with other features of the product that may be regulated by policies, including the label (e.g. nutritional claims or child-directed marketing strategies) or as well as price (e.g., taxes). A more comprehensive understanding of how these different features interact with nutrient warnings to influence behavior will be important for informing policy. 

## 5. Conclusions

This scoping review found that many experimental studies on FoP nutrient warnings focused on outcomes such as comprehension and behavioral intention. The studies found that while FoP nutrient warnings contain less detailed information than other FoP labeling systems, the warnings were visually attended to by consumers, easy to understand, helped consumers identify products high in nutrients of concern, and discouraged consumers from purchasing these products. However, considerable gaps in the evidence remain, particularly in the areas of negative affect and social interactions. Moreover, while our conceptual model and the existing literature measure important factors on the pathway from nutrient warning exposure to dietary behavioral change, additional elements of the food labeling regulation as well as consumer-level factors such as prior nutritional knowledge or socio-economic status may also influence the effectiveness of these warnings. Thus, more research will be needed to understand how nutrient warnings interact with other food environment and consumer-level factors to ultimately reduce SSB and ultra-processed food purchases. 

## Figures and Tables

**Table 1:** Study characteristics ^1^.

	% ^2^	*n*
**Region**		
Latin America	55%	12
US/Canada	32%	7
UK/Europe/Australia/New Zealand	32%	7
Asia	5%	1
**Setting**		
Online	64%	14
Laboratory	18%	4
Retail store	5%	1
School	18%	4
**Age Group**		
Children (≤13 years)	18%	4
Adolescents (13–18 years)	18%	4
Adults (18+ years)	91%	20
**Sex** (average % female) ^3^		
Children (≤13 years)	49% (±1%)	
Adolescents and adults	61% (±13%)	
**Education**		
Reported educational attainment	68%	15
Reported school type (public or private)	18%	4
% examining education as modifier/stratifier	14%	3
**Nutrient Warnings Shape**		
Rectangle	9%	2
Circle	14%	3
Octagon	77%	17
Triangle	18%	4
Magnifying glass w/exclamation	5%	1
Other	9%	2
**Nutrient Included in Warning, %**		
Sugar	91%	20
Saturated fat	55%	12
Total fat	14%	3
Sodium	59%	13
Calories	32%	7
Other	9%	2
**Outcome Category, %**		
Attention	23%	5
Comprehension	50%	11
Cognitive elaboration and message acceptance	36%	8
Negative affect and risk perception	18%	4
Behavioral intentions	41%	9
Behavioral response	23%	5
Other	14%	3
**Comparison FoP Label %**		
Multiple traffic light label	59%	13
Health Star Rating	41%	9
Guideline Daily Amount (or similar)	32%	7
Nutri-score	23%	5
Health warnings (graphic or text)	23%	5
Control (no FoP label or neutral label)	54%	12

^1^ All studies published in English-language journals between 1 January 2014 and 1 September 2019. ^2^ Average of averages (what was the mean % female across the studies). ^3^ % may not round to 100 for categories that are not mutually exclusive (e.g., a study tests labels on a beverage and a cereal or takes place in multiple countries).

**Table 2:** Study information: population, design, and outcomes.

Study	Setting	Population	Design	Outcomes
**Bollard et al., 2016** [59]	Online	New Zealand Adolescents/young adults age 13–24 years; *n* = 604	2 × 3 × 2 between-group: randomized to 1 of 3 FoP labels Control: no FoP label	Attitudes towards the product Social norms: Perceptions of a peer if they were drinking from the displayed can Attitudes towards policy Behavioral intentions: intentions to purchase.
**Arrúa et al., 2017** [60]	School	Montevideo, Uruguay Children age 8–13 years; *n* = 442;	Between-person: randomized to 1 of 2 FoP labels Control: no FoP label (within-person)	Behavioral intentions: children’s choice of product (images of product).
**Neal et al., 2017** [61]	Stores	Australia Adults age 18+ years; *n*= 1578	Between-person: randomized to 1 of 4 FoP labels Control: Nutrition information panel	Behavior: nutrient profile of food purchases. Elaboration and message acceptance: usefulness of the label; usefulness of having the label printed on every package. Comprehension: ease of understanding the label; current nutrition knowledge.
**Acton and Hammond, 2018** [62]	Online	Canada Adolescents/young adults age 16–32 years; *n* = 1000	Between-person: randomized to 1 of 4 FoP labels Control: None	Elaboration and message acceptance: perceptions of whether the label is harsh enough Self-efficacy: assessment of being in control of making healthy decisions?
**Acton and Hammond, 2018** [63]	Lab	Canada Adolescents and adults age ≥16 years; *n* = 675	Between person: randomized to 1 of 4 FoP labels Control: no label	Behavior: purchase of beverage.
**Egnell et al., 2018** [64]	Online	Argentina, Australia, Bulgaria, Canada, Denmark, France, Germany, Mexico, Singapore, Spain, United Kingdom, and United States Adults age ≥18 years; *n* = 12,015	Between-person: randomized to 1 of 5 FoP labels Control: no FoP label (within-person)	Comprehension: ranking of products according to nutritional quality.
**Goodman et al., 2018** [65]	Online	Canada, United States, Australia, United Kingdom Adults age 18–64 years; *n* = 11,617	Between-person: randomized to 1 of 11 FoP labels Control: no FoP label	Comprehension: identification of whether a product contained high, moderate, or low amounts of sugar or saturated fat. Elaboration and message acceptance: selection of the best symbol for informing consumers that a product is “high in” saturated fat and sugar.
**Khandpur et al., 2018** [66]	Online	Brazil Adults age ≥18 years; *n* = 1607	Between-person: randomized to 1 of 2 FoP labels Control: no FoP label (within-person)	Visibility/attention: rating of visibility and attention. Comprehension: identification of products high in nutrients of concern; ability to identify healthy products, rating of products’ healthfulness. Message acceptance: rating of credibility, usefulness, and ease of use. Behavioral intentions: likelihood of purchasing this or similar product.
**Lima, Ares, and Deliza, 2018** [67]	Schools (children) Online (parents)	Rio de Janeiro, Brazil Children age 6–9 years and 9–12 years; stratified by public and private schools Adults age ≥18 years, *n* = 278	Between-person: randomized to 1 of 3 FoP labels Control: none	Comprehension: rating of product healthfulness. Attitudes towards product: rating of perceived ideal consumption by children.
**Machín et al., 2017** [68]	Online (simulated online grocery store)	Uruguay Adults age ≥18 years; *n* = 437	Between-person: randomized to 1 of 3 FoP labels Control: no label	Behavioral intentions: share of intended ultra-processed food purchases; healthfulness of intended purchases.
**Machín et al., 2018** [69]	Online (simulated online grocery store)	Uruguay Adults age ≥18 years;*n* = 1182	Between-person: randomized to 1 of 3 FoP labels Control: No FoP label	Behavioral intentions: healthfulness of intended food purchases.
**Acton et al., 2019** [70]	Lab	Canada Adolescents and adults age ≥13 years; *n* = 3584	Between-person: randomized to 1 of 5 FoP labels Control: no FoP label	Attention: noticing the FoP warning label. Behavior: healthfulness of beverage purchases.
**Ang, Agrawal, and Finkelstein, 2019** [71]	Online (simulated online grocery store)	Singapore Adults age ≥21 years; *n* = 512	Between-person: randomized to 1 of 3 FoP labels Control: no FoP label	Behavioral intentions: healthfulness of intended purchases.
**Grummon et al., 2019** [72]	Online	United States Adults age ≥18 years; *n* = 1360	Between subjects: randomized between 1 of 4 FoP labels Control: control FoP label	Perceived message effectiveness: rating of concern about health effects of, unpleasantness of, and discouragement from drinking beverages with added sugar. Affect: rating of thinking about the health problems caused by beverages with added sugar and how much the label made them feel scared. Comprehension: knowledge of health harms of SSB consumption.
**Khandpur et al., 2019** [73]	Online	Brazil adults (ages not stated) *n* = 2419	Between participants: randomized to 1 of 4 FoP labels Control: no FoP label	Attention: rating of label visibility. Comprehension: identification of products high in or higher in nutrients of concern; identification of products not high in nutrients of concern; identification of healthier product, rating of product healthfulness. Behavioral intentions: likelihood of buying a product. Message acceptance: perceptions of label effects on behavior, understanding, helpfulness, and visibility.
**Lima et al., 2019** [74]	School (children) Lab (parents)	Brazil Children age 6–12 years; *n* = 400 Adults age 18–65 years; *n* = 400	Between subjects: randomized to 1 of 2 FoP labels Control: within-person	Behavior: selection of product to consume (regular-sugar version, the slightly reduced sugar version, or the highly reduced sugar version).
**Lima et al., 2019** [75]	School	Rio de Janeiro, Rio Pomba, Brazil Children age 6–12 years; *n* = 492	Between-person: randomized to 1 of 3 FoP labels Control: none	Affect: rating of feelings when eating the product (e.g., selection of emojis with the corresponding expression).
**Machín et al., 2019** [76]	Lab	Uruguay Adults age ≥18 years; *n* = 199	Between-person: randomized to 1 of 2 FoP labels Control: no FoP label	Attention: fixations on nutritional warnings Behavior: selection of a snack.
**Egnell et al, 2019** [77]	Online	The Netherlands Adults age ≥18 years; *n* = 1032	Between-person: randomized to 1 of 5 FoP labels Control: no FoP label (within-person)	Comprehension: ranking of products according to their nutritional quality Message acceptance: ratings of liking, awareness, perceived cognitive workload. Behavioral intentions: likelihood of purchasing a product.
**Talati et al, 2019** [78]	Online	Argentina, Australia, Bulgaria, Canada, Denmark, France, Germany, Mexico, Singapore, Spain, the UK, and the USA Adults age ≥18 years; *n* = 12,015	Between-person: randomized to 1 of 5 FoP labels Control: no FoP label (within-person)	Attention: rating of whether label stands out. Comprehension: rating of whether label is easy to understand, took too long to understand, is confusing, and provides needed information. Message acceptance: participants rated how much they liked the label, trusted, the label, and whether label should be compulsory.
**Ares et al, 2018** [79]	Online	Uruguay Adults age ≥18 years; *n* = 892	Between-person: randomized to 1 of 4 FoP labels Control: no FoP label	Comprehension: rating of product healthfulness. Behavioral intentions: likelihood of purchasing product.
**Egnell et al, 2019** [80]	Online	Germany Adults age ≥18 years; *n* = 1000	Between-person: randomized to 1 of 5 FoP labels Control: no FoP label (within-person)	Comprehension: ranking of products according to their nutritional quality.

**Table 3:** Summary of study results.

Study	Results
**Bollard et al., 2016 **[59]	Attitudes: Nutrient warnings (vs. control) had a negative effect on product preferences. Graphic warnings impacted product preferences more than nutrient warnings. Attitudes towards policy: More participants (66%) agreed or strongly agreed that SSBs should carry a (text) nutrient warning compared to graphic warning labels (50%). Behavioral intentions: Graphic warning and nutrient warnings decreased likelihood of intentions to purchase SSBs.
**Arrúa et al., 2017 **[60]	Behavioral intentions: For both product types, nutrient warnings discouraged children’s choice of product more than traffic light labels did.
**Neal et al., 2017 **[61]	Behavior: Compared to the control label, nutrient warnings led to healthier food purchases, but Health Star Ratings and Daily Intake Guides did not. Elaboration and message acceptance: There were no differences in ratings of how useful the labels were. Health Star Ratings were rated as more useful to have printed on every food package than were nutrient warnings. Comprehension: There were no differences between the Health Star Rating and nutrient warnings for consumers’ perception of their current nutrition knowledge. There were no differences in how easy the labels were to understand.
**Acton and Hammond, 2018 **[62]	Elaboration and message acceptance: Across all label conditions, at least 88% of respondents indicated the labels were “about right” or “not harsh enough.” Participants viewing the Health Star Rating were more likely to rate the symbol as not harsh enough compared with those who viewed any of the three nutrient warnings. Self-efficacy: Across all label conditions, 83% reported that the labels made them feel more in control or neither more/less in control. Participants viewing the Health Star Rating were less likely to state that the symbol made them feel more in control compared with those who viewed any of the three nutrient warnings.
**Acton and Hammond, 2018 **[63]	Behavior: There was no statistically significant effect of labeling, though there was a trend for the “high sugar” nutrient warnings to reduce the likelihood to purchase a sugary drink and encourage participants to purchase drinks with less sugar.
**Egnell et al., 2018 **[64]	Comprehension: All labels improved the number of correct responses in the ranking task. Nutri-score elicited the largest increase in the number of correct responses, followed by the multiple traffic light label, the Health Star Rating, nutrient warnings, and the reference intakes.
**Goodman et al., 2018 **[65]	Comprehension: Participants who viewed the red stop sign, caution triangle and exclamation mark, red circle, or magnifying glass + exclamation mark with high-in text were more likely to correctly identify the cereal as high-in saturated fat and sugar compared to those who saw the no-FoP control, with the highest odds observed among participants who viewed the red stop sign with the text “high-in” and the caution triangle, exclamation mark, and the text “high in.” Across all designs, respondents who viewed nutrient warnings with “high in” text had greater odds of responding correctly. Elaboration and message acceptance: Participants most often selected the red stop sign as the best nutrient warning symbol for informing consumers, followed by the triangle and exclamation mark.
**Khandpur et al., 2018 **[66]	Visibility/attention: Compared to participants who viewed traffic light labels, participants who viewed nutrient warnings rated the labels as having higher visibility and drawing more attention. Comprehension: Compared to when they viewed the no-label control, participants who viewed products with nutrient warnings improved their ability to identify products with excess nutrient content, improved their ability to identify healthy products, and decreased their perceptions of product healthfulness more than those who viewed traffic light labels. Behavioral intentions: Relative to when they viewed the no-label control condition, compared to participants who viewed traffic light labels, the participants who viewed products with nutrient warnings were more likely to express intent to buy the healthier product or neither products. Message acceptance: Compared to participants who viewed traffic light labels, participants who viewed nutrient warnings rated the labels as having higher credibility, usefulness, and ease of use.
**Lima, Ares, and Deliza, 2018 **[67]	Comprehension: There was no effect of labels on 6–9-year-old children or 9–12-year-old children from public schools. For 9–12-year olds from private schools, children who viewed nutrient warnings or traffic light labels rated the products as having lower healthfulness than children who viewed the GDA. Parents who viewed nutrient warnings rated the products as having lower healthfulness than parents who viewed the GDA. There were no differences in healthfulness ratings for the traffic light condition. Attitudes towards product: For parents with children in public schools, parents who viewed nutrient warnings rated products as having a lower ideal consumption frequency for specific products, including gelatin (compared to parents who viewed the GDA label) and corn snacks (compared to parents viewed the traffic light label).
**Machín et al., 2017 **[68]	Behavioral intentions: Overall, there were no differences between labeling conditions for mean share of intended ultra-processed food purchases or in mean nutrient content of intended food purchases. Participants in the nutrient warning condition decreased intended purchases of sweets and desserts.
**Machín et al., 2018 **[69]	Behavioral intentions: Compared to the control group, participants in both the nutrient warning condition and traffic light label condition decreased the average purchased density of calories, sugars, and saturated fats. Sodium density of purchases was also significantly decreased in the nutrient warning group compared to the control. Compared to the control group, participants in both the nutrient warning condition and traffic light label condition intended to purchase lower total amounts of calories, sugar, saturated fat, and sodium, though there were no statistically significant differences between the traffic light label and nutrient warning conditions. Compared to the control group, participants in both the nutrient warning condition and traffic light label condition purchased fewer products that were high in at least one nutrient. Compared to the control group, participants in both the nutrient warning condition and traffic light label condition spent less money on products in the categories: juice, cheese, bouillon cubes, spices, cereal bars, crackers, sweet cookies, cocoa, cream cheese, yogurt, nuts, jams, and ice creams. Participants in the traffic light label condition also decreased expenditures on oils.
**Acton et al., 2019 **[70]	Attention: A higher proportion of participants noticed the nutrient warnings than did participants in other labeling conditions. Behavior: For beverages, participants in the nutrient warning condition purchased beverages containing less sugar, saturated fat, and calories compared to those in the no-label control. There were no significant differences in the amount of sodium purchased between conditions. For foods, participants in the nutrient warning condition and traffic light label condition purchased foods with less sodium and fewer calories compared to the no label condition. Participants in the traffic light label condition also purchased less sodium and calories than those in the nutrition grade condition. Participants who viewed the Health Star Rating purchased fewer calories than those in the no label control condition. There were no significant differences in the amount of sugar or saturated fat purchased between conditions.
**Ang, Agrawal, and Finkelstein, 2019 **[71]	Behavioral intentions: Participants in the nutrient warning and health warning label conditions purchased a lower proportion of high-in-sugar products than those in the control, but this was statistically significant only for the health warnings. There were no differences between the nutrient warning and health warning groups. There were no differences between any group for total sugar purchased, sugar purchased per dollar spent, total spending, or total expenditure on high-sugar products. Results restricted to beverage only followed the same pattern as for total purchases.
**Grummon et al., 2019 **[72]	Perceived message effectiveness: Warnings that included health effects were perceived as more effective than those without health effects. Nutrient warnings were perceived as more effective than those without, though the effect was not as strong as for health warnings. Perceived message effectiveness was higher for warnings that included the marker word vs. those that did not, and for those that displayed an octagon vs. a rectangle-shaped label. Affect: Health effects had the biggest impact on thinking about harms and fear; nutrient disclosures also increased thinking about harms and fear. Thinking about harms and fear was higher for warnings that included the marker word vs. those that did not, and for those that displayed an octagon vs. a rectangle-shaped label. Comprehension: Nutrient warnings did not impact knowledge of health effects of SSB consumption. Health warnings increased knowledge that SSB intake leads to tooth decay, had no effect on knowledge that SSBs contribute to obesity or diabetes, and led to lower knowledge that SSBs contribute to heart disease.
**Khandpur et al., 2019 **[73]	Attention: Participants who viewed a nutrient warning triangle with the text “a lot of” rated the labels as more visible than participants who viewed the nutrient warning octagon. Comprehension: Participants in all nutrient warning conditions had higher scores for identifying nutrients in excess compared to participants in the control arm. Participants in the triangle “high in” condition correctly identified the most nutrients in excess. There were no differences between the nutrient warning conditions and the control for identifying nutrients not in excess. Participants in the triangle “high in” condition were the most likely to correctly select the product of a pair with the higher content of a nutrient of concern. Participants in the triangle “high in” condition and the triangle “a lot of” condition were most likely to correctly identify the overall healthier product out of the pair. Participants in all nutrient warning conditions had higher mean perceptions of levels of nutrients of concern than the control, and there were no differences between nutrient warnings. Participants in the triangle “high in” had the highest mean perception of nutrient levels. Participants in all nutrient warning conditions rated products as less healthy than the control, with no differences between types of nutrient warnings. Participants in the triangle “high in” had the lowest mean ratings of healthfulness. Behavioral intentions: Participants in all nutrient warning conditions expressed lower intentions to purchase than did participants in the control condition. Participants in the triangular “high in” condition expressed the lowest intentions to purchase. Message acceptance: Participants in all nutrient warning conditions had similar opinions on the effects of labels on improving purchasing and eating behaviors, the labels’ helpfulness, credibility, and ease of understanding.
**Lima et al., 2019 **[74]	Behavior: For adults and children, there was no effect of label type on selection of chocolate milk sample overall or by scenario (blind, expected, informed). For children, there was also no effect of label type on selection of grape nectar sample overall or by scenario. For adults, there was no effect on selection of grape nectar sample overall or by condition, except in the expected condition. In the expected condition, adults who were randomized to the nutrient warning condition were more likely to choose the highly reduced sugar sample (i.e., the only sample without a warning label) than were participants in the traffic light condition.
**Lima et al., 2019 **[75]	Affect: Children who were randomized to the nutrient warning and traffic light label conditions used emojis associated with positive emotions less frequently than children who were randomized to the GDA condition. The nutrient warning tended to have a greater effect on emoji use than the traffic light label. For some emojis, children from public schools tended to show greater changes in emoji use in response to the nutrient warning and traffic light label.
**Machín et al., 2019 **[76]	Attention: In total, 50% of the participants who were randomized to see the nutrient warning fixated their gaze on the warning for at least one product. Behavior: Participants in the nutrient warning condition were less likely to select products with excessive content of at least one nutrient, compared to the control group.
**Egnell et al., 2019 **[77]	Comprehension (objective understanding): Relative to the reference intakes, across all food categories, participants in the Nutri-score condition increased their ability to correctly rank the healthfulness of products the most compared to the no-label control condition. Participants in the nutrient warning and traffic light label conditions increased their ability to correctly rank cakes. Message acceptance: There were few differences in overall perceptions of labels. Behavioral intentions: Relative to the reference intake, there was no association between label type and change in nutritional quality of the product participants intended to purchase compared to the no label condition, overall and by food category. The exception was that participants in the nutrient warning condition were more likely to select a healthier breakfast cereal.
**Talati et al., 2019 **[78]	Attention: When asked to rate whether a label “did not stand out”, participants rated reference intakes the highest, followed by nutrient warning and Health Star Ratings. Nutri-score scored the lowest for not standing out. Comprehension: Participants rated nutrient warnings the lowest for “taking too long to understand,” and reference intakes the highest. Participants rated nutrient warnings as the highest for “easy to understand,” while Nutri-score scored the lowest. Participants scored traffic light labels the highest on providing all the information that they need, while Nutri-score was scored the lowest and nutrient warnings the second-lowest. Message acceptance: Participants rated the traffic light label the highest for liking and trust, with nutrient warnings scoring the lowest and second lowest on these items, respectively. Participants scored the traffic light label highest for being compulsory; Nutri-score scored the lowest and nutrient warnings the second-lowest.
**Ares et al., 2018 **[79]	Comprehension: The nutrient warnings had the greatest impact on perceptions of healthfulness and reduced perceived healthfulness compared to the control for cereals, yogurt, orange juice, bread, and mayonnaise. The Health Star Rating had the lowest impact on healthfulness perceptions. Behavioral intentions: Nutrient warnings had the greatest impact on purchase intentions, leading to decreased intentions to purchase breakfast cereals, yogurt, bread, and mayonnaise, compared to the control. Nutri-score reduced intentions to purchase only breakfast cereals and mayonnaise, while the Health Star Rating did not impact purchase intentions for any products.
**Egnell et al., 2019 **[80]	Comprehension: All labels improved the percentage of correct answers in the ranking exercise compared to the no-label control. Across categories, nutrient warnings were not associated with an increased likelihood in ability to correctly rank products, with the exception that nutrient warnings increased this likelihood for the cake category. Nutri-score was associated with the highest increase in ability to correctly rank products.

## Supplementary Materials

The following are available online at https://www.mdpi.com/2072-6643/12/2/569/s1, Table S1: PRISMA Checklist, Table S2: Search terms and hits, Table S3: Warning label designs, Table S4: Comparison labels, and Table S5: Study information.
