# Supplementary material for: Experimental Studies of Front-of-Package Nutrient Warning Labels on Sugar-Sweetened Beverages and Ultra-Processed Foods: A Scoping Review
Source: Nutrients. 2020 Feb 22;12(2):569. doi: 10.3390/nu12020569 (PMC7071470; doi:10.3390/nu12020569)
Supplement: Supplementary file 1 [file nutrients-12-00569-s001.zip › Supplements Revised/Table_S2_Search_terms_and_hits.docx]

**Table S2.** Search terms and hits

| **Database** | **Hits** | **Search String** |
| --- | --- | --- |
| **PubMed** | 175 | ((warning*[title/abstract] OR label*[title/abstract]) AND (pack*[title/abstract] OR FOP[title/abstract]) AND (food*[title/abstract] OR beverage*[title/abstract] OR drink*[title/abstract] OR snack*[title/abstract] OR nutrient*[title/abstract] OR nutrition*[title/abstract) AND (random*[title/abstract] OR trial*[title/abstract] OR experiment*[title/abstract])) AND (("2014/01/01"[PDat] : "3000/12/31"[PDat]) AND English[lang]) |
| **EBSCOhost (PsycInfo + CINAHL Plus)** | 177 | TI ( (warning* OR label*) AND (pack* OR FOP) AND (food* OR beverage* OR drink* OR snack* OR nutrient* OR nutrition*) AND (random* OR trial* OR experiment*) ) OR SU ( (warning* OR label*) AND (pack* OR FOP) AND (food* OR beverage* OR drink* OR snack* OR nutrient* OR nutrition*) AND (random* OR trial* OR experiment*) ) OR AB ( (warning* OR label*) AND (pack* OR FOP) AND (food* OR beverage* OR drink* OR snack* OR nutrient* OR nutrition*) AND (random* OR trial* OR experiment*) )  *Limiters:* Published Date: 20140101-; Peer Reviewed; Language: English |
| **Scopus** | 575 | TITLE-ABS-KEY ( ( warning* OR label* ) AND ( pack* OR fop ) AND ( food* OR beverage* OR drink* OR snack* OR nutrient* OR nutrition* ) AND ( random* OR trial* OR experiment* ) ) AND PUBYEAR > 2013 AND ( LIMIT-TO ( LANGUAGE , "English" ) ) AND ( LIMIT-TO ( SRCTYPE , "j" ) ) |
| **Web of Science** | 299 | (TI=((warning* OR label*) AND (pack* OR FOP) AND (food* OR beverage* OR drink* OR snack* OR nutrient* OR nutrition*) AND (random* OR trial* OR experiment*)) OR TS=((warning* OR label*) AND (pack* OR FOP) AND (food* OR beverage* OR drink* OR snack*) AND (random* OR trial* OR experiment*))) AND LANGUAGE: (English)  *Timespan:* 2014-2019. |
