# Supplementary material for: Experimental Studies of Front-of-Package Nutrient Warning Labels on Sugar-Sweetened Beverages and Ultra-Processed Foods: A Scoping Review
Source: Nutrients. 2020 Feb 22;12(2):569. doi: 10.3390/nu12020569 (PMC7071470; doi:10.3390/nu12020569)
Supplement: Supplementary file 1 [file nutrients-12-00569-s001.zip › Supplements Revised/Table_S3_Warning_label_designs.pdf]

Table S3. Warning label designs

OCTAGON

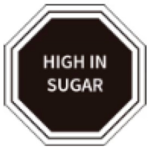

Egnell et al. [63,76,79]
Talati et al. [77]

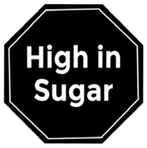

Ang, Agrawal, & Finkelstein [70]

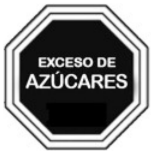

Ares et al. [78]

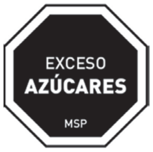

Machín et al. [75]

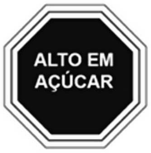

Arrúa et al. [59]
Lima et al. [66,73,74]

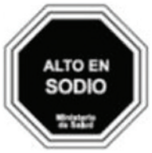

Machín et al. [67,68]

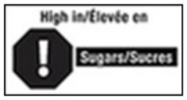

Acton & Hammond [61]

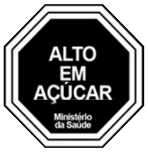

Khandpur et al. [72]

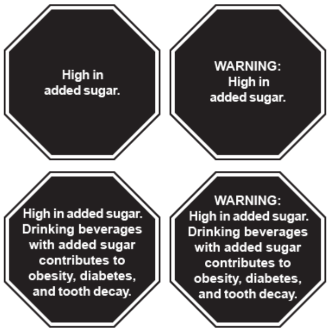

Grummon et al. [71]

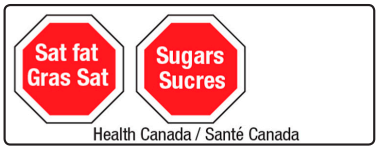

Goodman et al. [64]
Also tested with "High in" text (not shown)

(not shown) Black octagonal symbol with **"WARNING: high sugar content"** text
Bollard et al. [58]

RECTANGLE

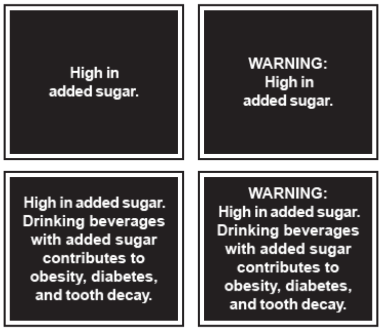

Grummon et al. [71]

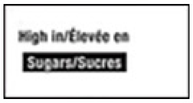

Acton & Hammond [61]

CIRCLE

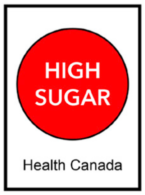

Acton & Hammond [62]

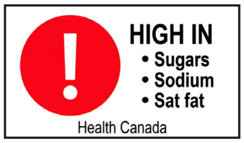

Acton et al. [69]

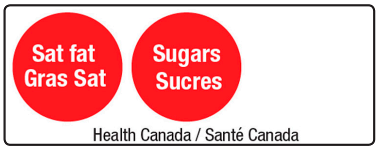

Goodman et al. [64]
Also tested with "High in" text (not shown)

TRIANGLE

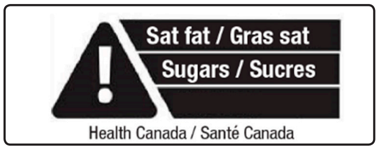

Goodman et al. [64]
Also tested with "High in" text (not shown)

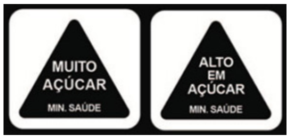

Khandpur et al. [72]

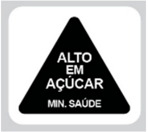

Khandpur et al. [65]

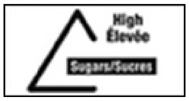

Acton & Hammond [61]

OTHER

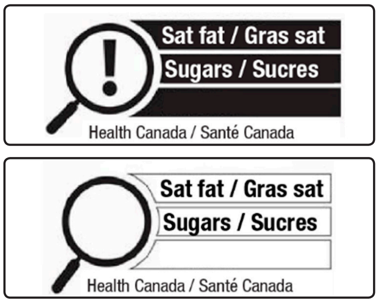

Goodman et al. [64]
Also tested with "High in" text (not shown)

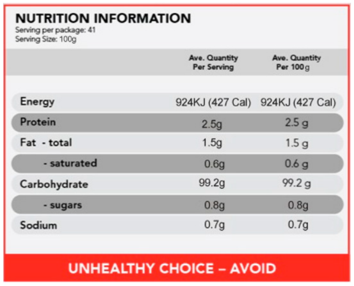

Neal et al. [60]
