# Supplementary material for: Experimental Studies of Front-of-Package Nutrient Warning Labels on Sugar-Sweetened Beverages and Ultra-Processed Foods: A Scoping Review
Source: Nutrients. 2020 Feb 22;12(2):569. doi: 10.3390/nu12020569 (PMC7071470; doi:10.3390/nu12020569)
Supplement: Supplementary file 1 [file nutrients-12-00569-s001.zip › Supplements Revised/Table_S4_Comparison_labels.docx]

| **Table S4.** Comparison labels | | | | | | | |  |  |  |  |  |
| --- | --- | --- | --- | --- | --- | --- | --- | --- | --- | --- | --- | --- |
|  | **Multiple Traffic light** | **Health Star Rating** | | **GDAs, DIGs, RIs Facts up Front** | **Nutri-Score** | **Others** | **Control** |  |  |  |  |  |
|  | Interpretive, multiple nutrients, color-coded | Summary, single color, graded | **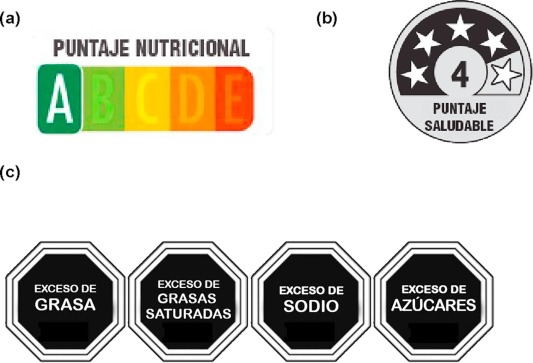** | Informative, multiple nutrients, single color | Summary, graded,  color-coded | Health text or graphic image warnings | No FoP label or neutral label |  |  |  |  |  |
| **Examples**  *Actual designs vary* | *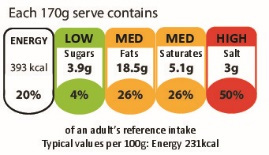* | 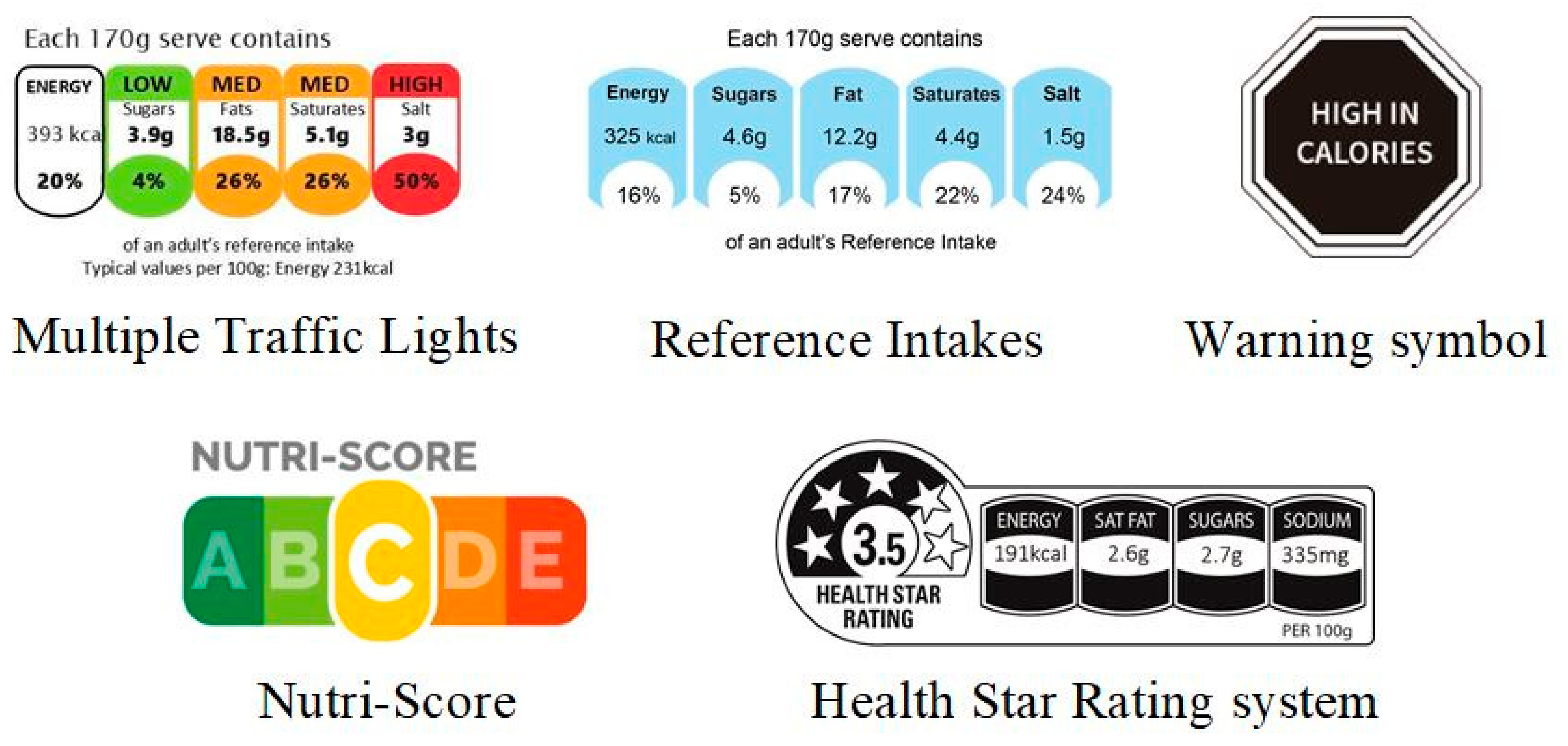 | | **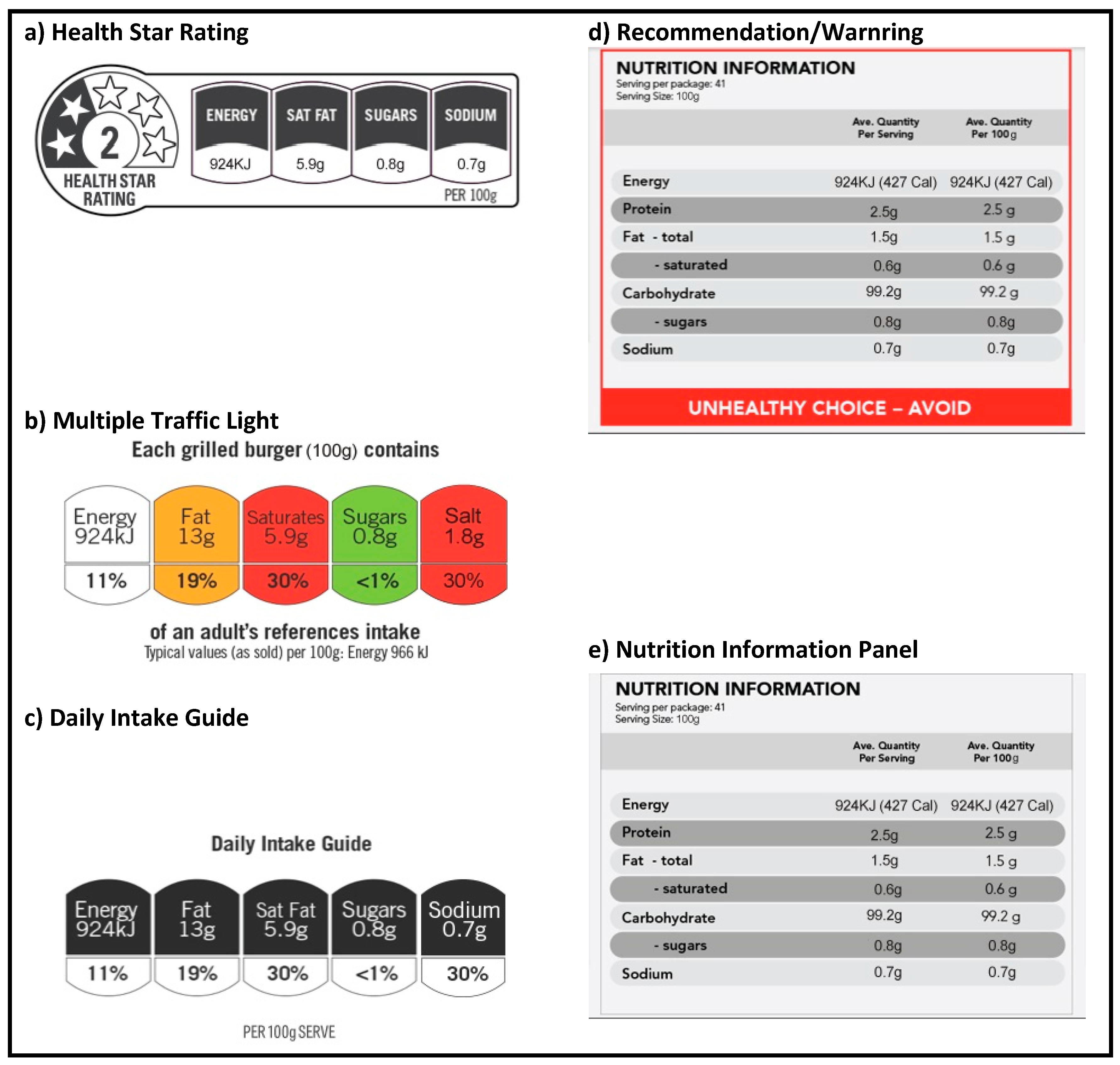** | 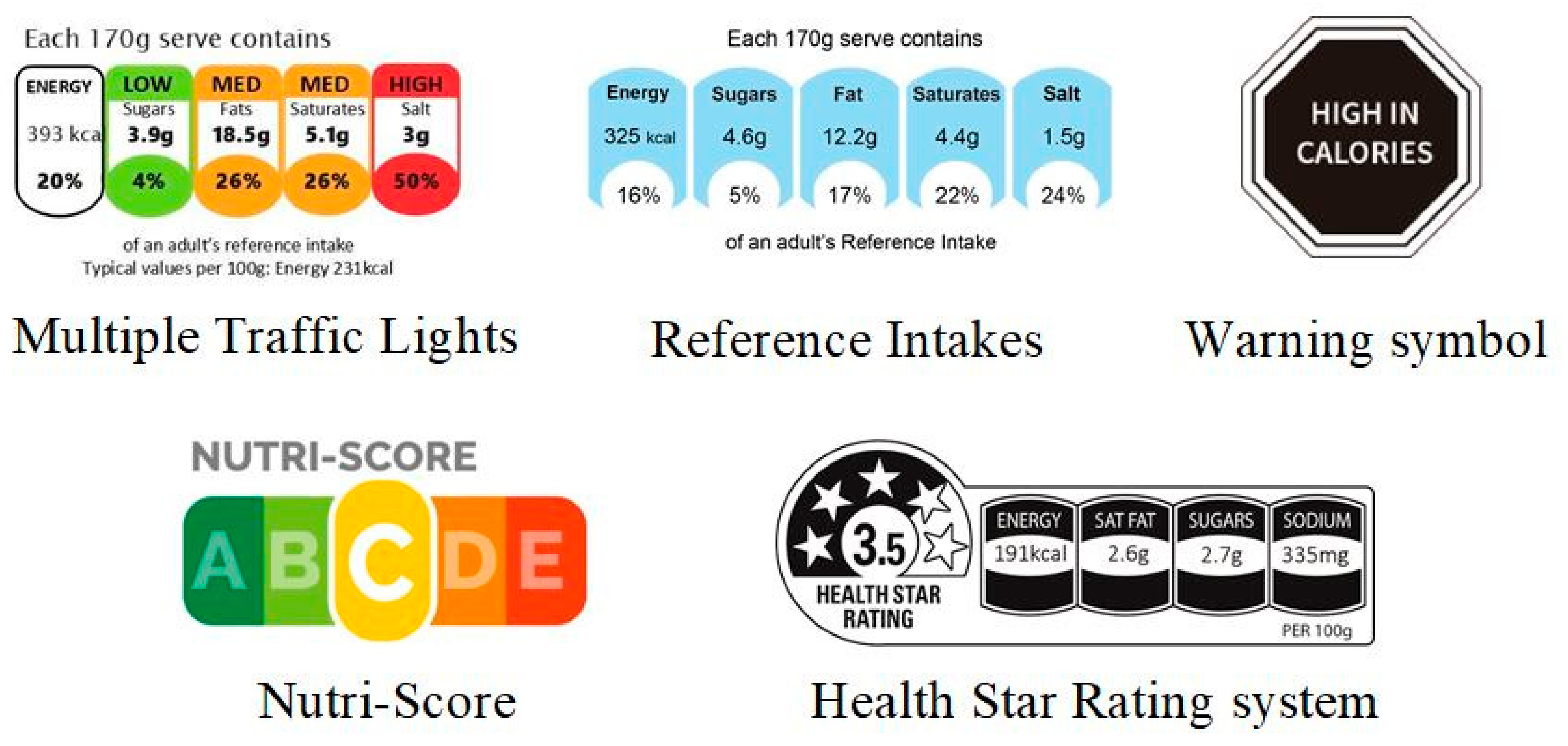 | **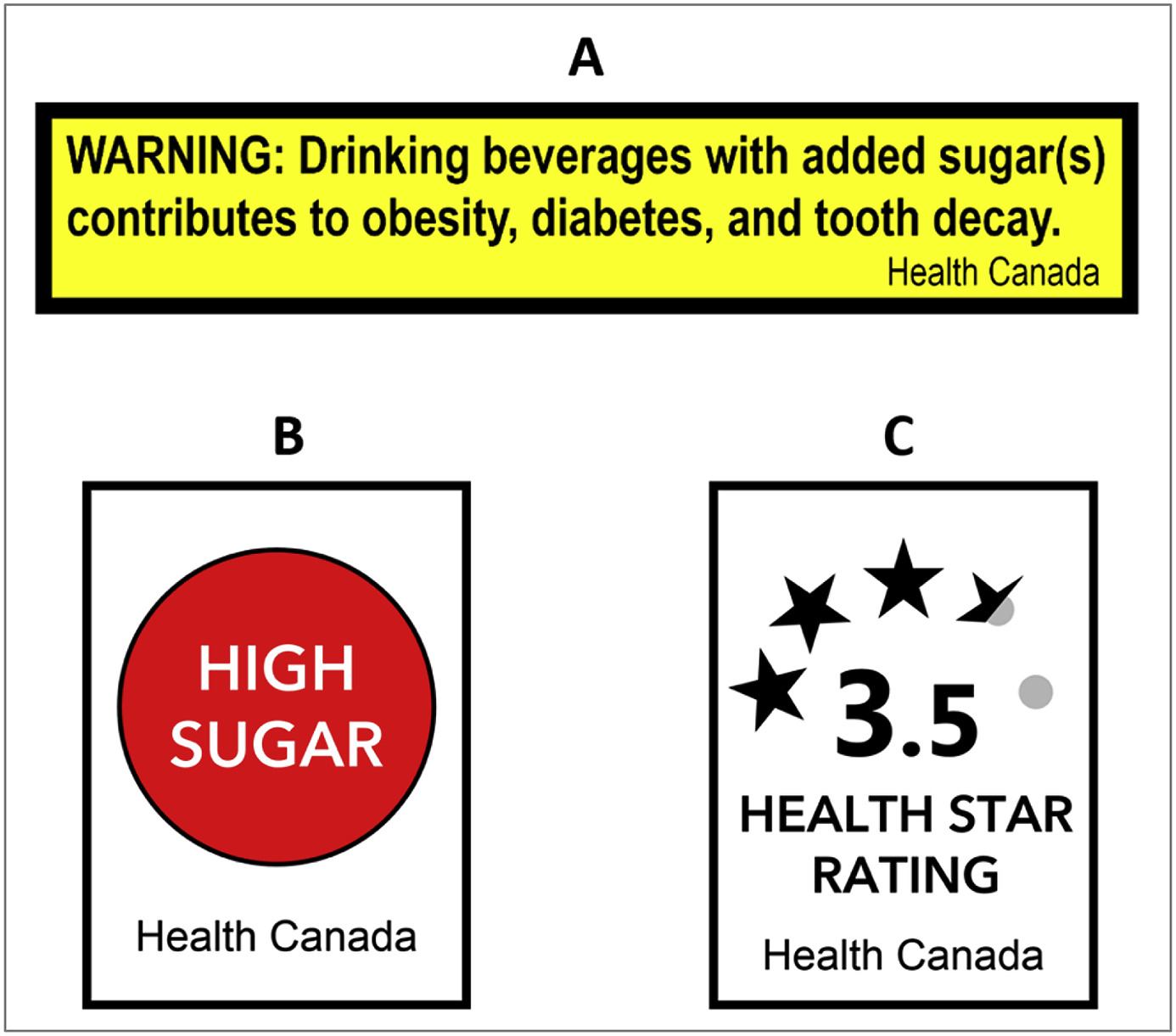** | N/A |  |  |  |  |  |
| **Bollard et al., 2016 [1]** |  |  | |  |  |  |  |  |  |  |  |  |
| **Arrúa et al., 2017 [2]** |  |  | |  |  |  |  |  |  |  |  |  |
| **Neal et al., 2017 [3]** |  |  | |  |  |  |  |  |  |  |  |  |
| **Acton & Hammond, 2018 [4]** |  |  | |  |  |  |  |  |  |  |  |  |
| **Acton & Hammond, 2018 [5]** |  |  | |  |  |  |  |  |  |  |  |  |
| **Egnell et al., 2018 [6]** |  |  | |  |  |  |  |  |  |  |  |  |
| **Goodman et al., 2018 [7]** |  |  | |  |  |  |  |  |  |  |  |  |
| **Khandpur et al., 2018 [8]** |  |  | |  |  |  |  |  |  |  |  |  |
| **Lima, Ares, & Deliza, 2018 [9]** |  |  | |  |  |  |  |  |  |  |  |  |
| **Machín et al., 2017 [10]** |  |  | |  |  |  |  |  |  |  |  |  |
| **Machín et al., 2018 [11]** |  |  | |  |  |  |  |  |  |  |  |  |
| **Acton et al., 2019 [12]** |  |  | |  |  |  |  |  |  |  |  |  |
| **Grummon et al., 2019 [13]** |  |  | |  |  |  |  |  |  |  |  |  |
| **Khandpur et al., 2019 [14]** |  |  | |  |  |  |  |  |  |  |  |  |
| **Lima et al., 2019 [15]** |  |  | |  |  |  |  |  |  |  |  |  |
| **Lima et al., 2019 [16]** |  |  | |  |  |  |  |  |  |  |  |  |
| **Machín et al., 2019 [17]** |  |  | |  |  |  |  |  |  |  |  |  |
| **Talati et al, 2019 [18]** |  |  | |  |  |  |  |  |  |  |  |  |
| **Ares et al, 2018 [19]** |  |  | |  |  |  |  |  |  |  |  |  |
| **Egnell et al, 2019 [20]** |  |  | |  |  |  |  |  |  |  |  |  |
| **Egnell et al, 2019 [21]** |  |  | |  |  |  |  |  |  |  |  |  |
| **Ang, Agrawal, & Finkelstein, 2019 [22]** |  |  | |  |  |  |  |  |  |  |  |  |
|  |  |  | |  |  |  |  |  |  |  |  |  |

*Black shading indicates that the study used this type of label.*

*Abbreviations: GDA (Guideline Daily Amount), DIGs (Daily Intake Guidelines), RI (Reference Intake), NFP (Nutrition Facts Panel).*

**References**

1. Bollard, T.; Maubach, N.; Walker, N.; Ni Mhurchu, C. Effects of plain packaging, warning labels, and taxes on young people's predicted sugar-sweetened beverage preferences: an experimental study. *International Journal of Behavioral Nutrition & Physical Activity* **2016**, *13*, 1-7, doi:10.1186/s12966-016-0421-7.

2. Arrúa, A.; Curutchet, M.R.; Rey, N.; Barreto, P.; Golovchenko, N.; Sellanes, A.; Velazco, G.; Winokur, M.; Giménez, A.; Ares, G. Impact of front-of-pack nutrition information and label design on children's choice of two snack foods: Comparison of warnings and the traffic-light system. *Appetite* **2017**, *116*, 139-146, doi:10.1016/j.appet.2017.04.012.

3. Neal, B.; Crino, M.; Dunford, E.; Gao, A.; Greenland, R.; Li, N.; Ngai, J.; Mhurchu, C.N.; Pettigrew, S.; Sacks, G., et al. Effects of different types of front-of-pack labelling information on the healthiness of food purchases—a randomised controlled trial. *Nutrients* **2017**, *9*, doi:10.3390/nu9121284.

4. Acton, R.B.; Hammond, D. Do Consumers Think Front-of-Package "High in" Warnings are Harsh or Reduce their Control? A Test of Food Industry Concerns. *Obesity* **2018**, *26*, 1687-1691, doi:10.1002/oby.22311.

5. Acton, R.B.; Hammond, D. The impact of price and nutrition labelling on sugary drink purchases: Results from an experimental marketplace study. *Appetite* **2018**, *121*, 129-137, doi:10.1016/j.appet.2017.11.089.

6. Egnell, M.; Talati, Z.; Hercberg, S.; Pettigrew, S.; Julia, C. Objective understanding of front-of-package nutrition labels: An international comparative experimental study across 12 countries. *Nutrients* **2018**, *10*, doi:10.3390/nu10101542.

7. Goodman, S.; Vanderlee, L.; Acton, R.; Mahamad, S.; Hammond, D. The impact of front-of-package label design on consumer understanding of nutrient amounts. *Nutrients* **2018**, *10*, doi:10.3390/nu10111624.

8. Khandpur, N.; de Morais Sato, P.; Mais, L.A.; Bortoletto Martins, A.P.; Spinillo, C.G.; Garcia, M.T.; Urquizar Rojas, C.F.; Jaime, P.C. Are front-of-package warning labels more effective at communicating nutrition information than traffic-light labels? A randomized controlled experiment in a Brazilian sample. *Nutrients* **2018**, *10*, doi:10.3390/nu10060688.

9. Lima, M.; Ares, G.; Deliza, R. How do front of pack nutrition labels affect healthfulness perception of foods targeted at children? Insights from Brazilian children and parents. *Food Quality and Preference* **2018**, *64*, 111-119, doi:10.1016/j.foodqual.2017.10.003.

10. Machín, L.; Arrúa, A.; Giménez, A.; Curutchet, M.R.; Martínez, J.; Ares, G. Can nutritional information modify purchase of ultra-processed products? Results from a simulated online shopping experiment. *Public Health Nutr* **2018**, *21*, 49-57, doi:10.1017/S1368980017001185.

11. Machín, L.; Aschemann-Witzel, J.; Curutchet, M.R.; Giménez, A.; Ares, G. Does front-of-pack nutrition information improve consumer ability to make healthful choices? Performance of warnings and the traffic light system in a simulated shopping experiment. *Appetite* **2018**, *121*, 55-62, doi:10.1016/j.appet.2017.10.037.

12. Acton, R.B.; Jones, A.C.; Kirkpatrick, S.I.; Roberto, C.A.; Hammond, D. Taxes and front-of-package labels improve the healthiness of beverage and snack purchases: a randomized experimental marketplace. *International Journal of Behavioral Nutrition & Physical Activity* **2019**, *16*, N.PAG-N.PAG, doi:10.1186/s12966-019-0799-0.

13. Grummon, A.H.; Hall, M.G.; Taillie, L.S.; Brewer, N.T. How should sugar-sweetened beverage health warnings be designed? A randomized experiment. *Prev Med* **2019**, *121*, 158-166, doi:10.1016/j.ypmed.2019.02.010.

14. Khandpur, N.; Mais, L.A.; Sato, P.D.; Martins, A.P.B.; Spinillo, C.G.; Rojas, C.F.U.; Garcia, M.T.; Jaime, P.C. Choosing a front-of-package warning label for Brazil: A randomized, controlled comparison of three different label designs. *Food Research International* **2019**, *121*, 854-861, doi:10.1016/j.foodres.2019.01.008.

15. Lima, M.; de Alcantara, M.; Ares, G.; Deliza, R. It is not all about information! Sensory experience overrides the impact of nutrition information on consumers’ choice of sugar-reduced drinks. *Food Quality and Preference* **2019**, *74*, 1-9, doi:10.1016/j.foodqual.2018.12.013.

16. Lima, M.; de Alcantara, M.; Martins, I.B.A.; Ares, G.; Deliza, R. Can front-of-pack nutrition labeling influence children's emotional associations with unhealthy food products? An experiment using emoji. *Food Research International* **2019**, *120*, 217-225, doi:10.1016/j.foodres.2019.02.027.

17. Machín, L.; Curutchet, M.R.; Giménez, A.; Aschemann-Witzel, J.; Ares, G. Do nutritional warnings do their work? Results from a choice experiment involving snack products. *Food Quality and Preference* **2019**, *77*, 159-165, doi:10.1016/j.foodqual.2019.05.012.

18. Talati, Z.; Egnell, M.; Hercberg, S.; Julia, C.; Pettigrew, S. Consumers’ Perceptions of Five Front-of-Package Nutrition Labels: An Experimental Study Across 12 Countries. *Nutrients* **2019**, *11*, 1934.

19. Ares, G.; Varela, F.; Machín, L.; Antúnez, L.; Giménez, A.; Curutchet, M.R.; Aschemann-Witzel, J. Comparative performance of three interpretative front-of-pack nutrition labelling schemes: Insights for policy making. *Food Quality and Preference* **2018**, *68*, 215-225.

20. Egnell, M.; Talati, Z.; Gombaud, M.; Galan, P.; Hercberg, S.; Pettigrew, S.; Julia, C. Consumers’ Responses to Front-of-Pack Nutrition Labelling: Results from a Sample from The Netherlands. *Nutrients* **2019**, *11*, 1817.

21. Egnell, M.; Talati, Z.; Pettigrew, S.; Galan, P.; Hercberg, S.; Julia, C. Comparison of front-of-pack labels to help German consumers understand the nutritional quality of food products. Color-coded labels outperform all other systems. *Ernahrungs Umschau* **2019**, *66*, 76-84.

22. Ang, F.J.L.; Agrawal, S.; Finkelstein, E.A. Pilot randomized controlled trial testing the influence of front-of-pack sugar warning labels on food demand. *BMC Public Health* **2019**, *19*, 1-8, doi:10.1186/s12889-019-6496-8.
