# Supplementary material for: Experimental Studies of Front-of-Package Nutrient Warning Labels on Sugar-Sweetened Beverages and Ultra-Processed Foods: A Scoping Review
Source: Nutrients. 2020 Feb 22;12(2):569. doi: 10.3390/nu12020569 (PMC7071470; doi:10.3390/nu12020569)
Supplement: Supplementary file 1 [file nutrients-12-00569-s001.zip › Supplements Revised/Table_S5_Study Info.docx]

**Table S5.** Study Information

| **Study** | **Setting*** | **Population** | **Design** | **Stimuli** | **Labels** | **Outcomes** |
| --- | --- | --- | --- | --- | --- | --- |
| **Bollard et al., 2016 [1]*** | Online | New Zealand  Adolescents/young adults age 13-24y; n=604; 51.0% female  Education level: 5.8% intermediate; 49.3% high school; 28.3% tertiary, 1.5% other, 16.4% not currently studying  Recruited from: Market research company panel of soft-drink consumers | **2x3x2 between-group:** Participants randomized to receive 1 of 3 labeling conditions  **Control:** No FoP label | SSB | 1. Control: no label 2. Nutrient text warning: Black octagonal symbol with “WARNING: high sugar content” text 3. Graphic warning: image of dental carries with text, “WARNING: consuming beverages with added sugar contributes to tooth decay.” | **Attitudes** towards the product:   - Self-reported attitudes: expensive/cheap, unattractive/ attractive, low quality/high quality, uncool/cool, un-healthy/ healthy, and tasted bad or good. - (social norms) Perceptions of a peer if they were drinking from the can displayed (boring/interesting, unpopular/ popular, unfashionable/fashionable, and old/young)   **Attitudes** towards policy  **Behavioral intentions:** Intentions to purchase |
| **Arrúa et al., 2017 [2]*** | **School** | Uruguay  Children age 8-13y; n=442; 50% female  Education: public schools  Recruited from: 12 public primary schools in Montevideo | **Between-person:** Participants randomly assigned to 1 of 2 label conditions  **Control:** No FoP label (within-person) | Wafer cookies and orange juice | 1. Traffic lights 2. Nutrient text warning: Black octagonal symbol with “High in sugar” text | **Behavioral intentions:** children’s choice of product (images of product) |
| **Neal et al., 2017 [3]** | Stores | Australia  Adults age 18y and older; n= 1578; 83.8% female  Education level: Primary/secondary (21.8%); tertiary (50.1%), post-graduate (27.4%); none of the above (0.7%)  Recruited from: Nationwide convenience sample | **Between-person:** Participants randomized to receive 1 of 4 labels viewed on a smartphone application while making food purchases | Packaged foods and beverages | 1. Health Star Rating 2. Daily intake guides 3. Nutrient text recommendations and warnings: all products showed nutrition information. Products in the lowest 20%/top 20% of nutrient profile score included text “UNHEALTHY CHOICE-AVOID” or “HEALTHY CHOICE,” respectively 4. Control (nutrition information panel) | **Behavior:** nutrient profile of food purchases  **Elaboration and Message Acceptance:** usefulness of the label; usefulness to have the label printed on every package***;**  **Comprehension:** ease of understanding the label, and current nutrition knowledge  *Outcomes only reported for labels vs. Health Star Rating* |
| **Acton & Hammond, 2018 [4]** | Online | Canada  Adolescents/young adults age 16-32 y; n=1000; 69.7% female  Education level: Not reported  Recruited from: National cohort study of youth and young adults, recruited from 5 cities (Edmonton, Halifax, Montreal, Toronto, Vancouver) | **Between-person:** Participants randomly assigned to 1 of 4 FoP label conditions  **Control:** none | Generic packaged beverage | 1. Nutrient text warning: “high in sugar,” no symbol or imagery 2. Nutrient-based text warning: Octagon symbol with “high in sugar” text 3. Nutrient text warning: Triangle with “high in sugar” text 4. Health Star Rating (modeled after Australia/New Zealand) | **Elaboration and Message Acceptance:** Do you think this label is harsh enough?  **Self-efficacy** (Control)**:** Would this label make you feel more in control of making healthy decisions? |
| **Acton & Hammond, 2018 [5]** | Laboratory | Canada  Adolescents and adults age ≥16y; n=675; 53.9% female  Education level: Not reported  Recruited from: Convenience sample recruited at a shopping mall in southwestern Ontario, Canada. | **Between person:** Participants randomized to one of 4 labeling conditions  **Control: N**o label | Packaged beverages | 1. No label 2. Health Star Rating 3. Nutrient text warning: Red circle symbol with “High sugar” text 4. Health text warning: “WARNING: Drinking beverages with added sugar(s) contributes to obesity, diabetes, and tooth decay. | **Behavior:** Purchase of beverage |
| **Egnell et al., 2018 [6]** | Online | Argentina, Australia, Bulgaria, Canada, Denmark, France, Germany, Mexico, Singapore, Spain, the United Kingdom (UK), and the United States  Adults age ≥18y; n=12,015; 50.0% female  Education level: Primary education (3.6%), secondary education (23.4%), trade certificate (21.4%), university (33.8%), post-graduate (17.7%)  Recruited from: International web panel provider (PureProfile) using quota sampling by age, sex, and income level. | **Between-person:** participants randomized to one of 5 labeling conditions  **Control:** No FoP label (within-person) | Packaged pizzas, cakes, and breakfast cereals. | 1. Health star rating 2. Multiple traffic lights 3. Nutri-score 4. Reference intakes 5. Nutrient text warning: Black octagon with text “High in [nutrient]” (sugar, calories, saturated fat, and sodium, depending on the product) | **Comprehension:** Participants ranked three sets of three products according to their nutritional quality (highest, medium, or lowest nutritional quality). |
| **Goodman et al., 2018 [7]** | Online | Canada, United States, Australia, United Kingdom  Adults age 18y-64y; n=11,617; 52.9% female  Education level: Low (22.8%), middle (27.7%), high (49.5%)  Recruited from: Nielsen Consumer Insights Global panel and partner panels. | **Between-person:** Participants randomized to one of 11 labeling conditions  **Control:** No FoP label | Breakfast cereal | 1. Control (no label) 2. Warning icon: Red circle 3. Warning icon: Red octagon 4. Warning icon: Magnifying glass 5. Warning icon: Magnifying class and exclamation mark, 6. Warning icon: Caution triangle and exclamation mark 7. Nutrient text warning: Red circle with “High in sugar” and “High in saturated fat” text 8. Nutrient text warning: Red octagon with “High-in” text 9. Nutrient text warning: Magnifying glass with “High-in” text 10. Nutrient text warning: Magnifying class and exclamation mark with “High-in” text 11. Text warning: Caution triangle and exclamation mark with “High-in” text | **Comprehension:** Participants identified whether a product contained high, moderate, or low amounts of sugar or saturated fat.  **Elaboration and Message Acceptance:** Participants selected the best symbol for informing consumers that a product is “high in” saturated fat and sugar. |
| **Khandpur et al., 2018 [8]** | Online | Brazil  Adults age ≥18y; n=1,607; 52.5% female  Education level: Primary or less (13.2%), secondary (68.9%), tertiary (17.9%)  Recruited from: Convenience sample from an online panel. | **Between-person:** Participants randomized to 1 of 2 labeling conditions  **Control:** No FoP label (within-person) | Savory snack, chocolate cookies, flavored lemonade (single comparison task); savory biscuits, instant soups, breakfast cereals (product comparison tasks) | 1. Traffic light label 2. Nutrient text warning: Black triangles with text “high in” sugar, saturated fat, total fat, or sodium and black triangles with text “Contains” *trans* fat and non-caloric sweetener. | **Visibility/attention:** Participants rated products on visibility and attention.  **Comprehension:** Participants indicated whether the product contains certain nutrients in levels higher than recommended for a healthy diet and rated the products’ healthfulness.  **Message acceptance:** Participants rated products on credibility, usefulness, and ease of use.  **Behavioral intentions:** Participants rated their likelihood of purchasing this product or similar product. |
| **Lima, Ares, & Deliza, 2018 [9]** | Schools (children)  Online (parents) | Brazil  Children age 6-9y and 9-12y; stratified by school type: private school (58%), public school (42%); n=318, 49% female  Adults age ≥18y, n=278; 83% female  Education level: Elementary school (4%), high school (27%), incomplete higher education (10%), higher education (34%), post-graduate (25%).  Recruited from: Private school in Rio de Janeiro (middle-/high-income); NGO that develops activities for low-income children from public schools. | **Between-person:** Participants randomized to one of 3 labeling conditions  **Control:** None | Chocolate milk, cookies, sponge cake, frosted cornflakes, gelatin, yogurt, fruit-flavored beverage, and corn snack. | 1. Daily Guideline Amounts (GDA) 2. Traffic lights 3. Nutrient text warning: Black octagons containing text, “High in” sugar, saturated fat, sodium, or calories. | **Comprehension:** Participants rated the healthfulness of the product.  **Attitudes towards product** (perceived ideal consumption): Participants rated how often, ideally, the product should be consumed by their children. |
| **Machín et al., 2017 [10]** | Online (simulated online grocery store) | Uruguay  Adults age ≥18y; n=437; 75% female  Education level: Primary school (6%), secondary school (55%), technical education (7%), university (22%), postgraduate (10%)  Recruited from: Consumer database and a Facebook advertisement. | **Between-person**: Participants randomized to one of 3 labeling conditions  **Control:** No label | 232 food and beverage products in 16 food categories, ranging from natural/minimally processed to ultra-processed | 1. Control: no FoP nutritional information 2. Traffic light label 3. Nutrient text warning: Black octagons containing text, “High in” sugar, saturated fat, sodium, or calories, depending on the product. | **Behavioral intentions:** share of intended ultra-processed food purchases as defined by number of products and expenditure on ultra-processed foods; mean calories, sugar, saturated fat, and sodium content of purchased items) |
| **Machín et al., 2018 [11]** | Online (simulated online grocery store) | Uruguay  Adults age ≥18y; n=1,182; 91% female  Education level: Primary school or less (6%), incomplete secondary (22%), secondary (32%), technical school (11%), incomplete university (20%), university or post-graduate (9%)  Recruited from: Online, from Facebook ad | **Between-person:** Participants randomized to one of 3 labeling conditions  **Control:** No FoP label | 232 food and beverage products in 16 food categories, | 1. Control: no FoP nutrition label 2. Traffic light label 3. Nutrient text warning: Black octagons containing text, “High in” sugar, saturated fat, sodium, or calories, depending on the product. | **Behavioral intentions:** healthfulness of intended food purchases **(**energy density, sugar density, saturated fat density, and sodium density; total content of calories, sugar, saturated fat, and sodium; and number of products with high-in content of calories, sugar, saturated fat, and sodium) |
| **Acton et al., 2019 [12]** | Laboratory | Canada  Adolescents, adults age ≥13y; n=3,584; 56% female  Education level: High school or less (26.6%), trade school or vocational/general college (11.7%), University (61.7%)  Recruited from: Convenience sample from large shopping centers in 3 Ontario cities (Kitchener, Waterloo, and Toronto) | **Between-person:** Participants randomized to one of 5 labeling conditions  **Control:** No FoP label | Images of 20 packaged beverages and 20 snack foods (including chips, candies, cookies and granola bars, fruit, and others) | 1. Control: no front-of package label 2. Nutrient text warning: A red circle containing a white exclamation mark with the text “High in” sugars, sodium, and/or saturated fat 3. Traffic light label 4. Health Star Rating (modeled after Australia and New Zealand) 5. Nutrition Grade (modeled after Nutri-score): color-coded rating from A (healthy) to E (least healthy) | **Attention: N**oticing the FoP warning label.  **Behavior:** Healthfulness of beverage purchases **(**mean sugar, calories, sodium, and saturated fat purchased) |
| **Ang, Agrawal, & Finkelstein, 2019 [13]** | Online  (simulated online grocery store) | Singapore  Adults age ≥21y; n=512; 46.7% female  Education level: Not reported  Recruited from: Online panel | **Between-person:** Participants randomized to one of 3 labeling conditions  **Control: N**o FoP label | 1800 non-perishable food and beverage products | 1. Control: no front-of package label 2. Nutrient text warning: black octagon with the text “High in Sugar” 3. Text-based health warning with text: HEALTH WARNING: Consuming products with added sugar(s) contributes to obesity, diabetes, and tooth decay. | **Behavioral intentions: H**ealthfulness of intended purchases (proportion of high-in-sugar products purchased; total sugar purchased per trip, sugar purchased per dollar spent, total spending, and total expenditure on high-in-sugar products). |
| **Grummon et al., 2019 [14]** | Online | United States  Adults age ≥18y; n=1,360; 47% female  Education level: High school or less (13%), some college (23%), college or associates (52%), or postgraduate (13%)  Recruited from: National convenience sample from Amazon Mechanical Turk | **Between subjects:** participants randomized between one of 4 labeling conditions  **Control:** Text-only: “Always read the nutrition facts panel.” | Packaged beverage | 1. Control: Always read the Nutrition Facts Panel 2. Nutrient text warning: Black octagon or rectangle with text “Drinking beverages with added sugar contributes to obesity, diabetes, and tooth decay” 3. Nutrient text warning: Black octagon or rectangle with text “High in added sugar” 4. Health and nutrient text warning: Black octagon or rectangle with text: “High in added sugar. Drinking beverages with added sugar contributes to obesity, diabetes, and tooth decay.” | **Perceived message effectiveness:** Participant rated how much the label makes them concerned about the health effects of drinking beverages with added sugar, makes drinking these beverages seem unpleasant, and discourages them from drinking these beverages.  **Affect:** Participants rated how much the label made them think about the health problems caused by beverages with added sugar and how much the label made them feel scared.  **Comprehension**: Knowledge of health harms of SSB consumption. |
| **Khandpur et al., 2019 [15]** | Online | Brazil  Adults (ages not stated); n=2,419; 59.5% female  Education level: Primary or less (4%), secondary (79%), and tertiary (17%)  Recruited from: Convenience sample from an online panel. | **Between participants:** participants randomized to one of four labeling conditions.  **Control:** a control condition with no FoP label | Section 1: a cereal bar, crackers, and chocolate milk  Section 2: breakfast cereals, breads and juices | 1. Control: no label 2. Nutrient text warning: Triangle with text “A lot of” 3. Nutrient text warning: Triangle with text “High in” 4. Nutrient text warning: octagon with text “High In”   For all warning label arms, nutrients disclosed included free sugars, saturated fat, total fat, or sodium or whether the product contained *trans* fat or artificial sweeteners | **Attention:** Participants rated label visibility.  **Comprehension:** Participants rated:1) whether product contains certain nutrients in levels higher than recommended for a healthier diet; and 2) whether product contains certain nutrients not in excess (within recommended levels).They also selected which of two products had a larger quantity of nutrients and which of two products was relatively healthier. Participants rated how much of a nutrient is in one portion of the product and the healthfulness of the product.  **Behavioral intentions:** Participants rated likelihood of buying a product or which of a pair of products they would buy.  **Message acceptance:** Perceived effects on behavior, understanding, helpfulness, and visibility. |
| **Lima et al., 2019 [16]** | School (children)  Lab  (parents) | Brazil  Children age 6-12 y; n=400; 48% female Adults age 18y-65y; n=400; 61% female  Education: Private schools (children); not reported (adults)  Recruited from: Private schools (children) and a supermarket (adults) in Rio de Janeiro | **Between subjects:** Participants randomized to one of 2 labeling conditions  **Control:** Within-person, participants exposed to control *(3 versions of product presented in cups without packaging for participants to taste)*, expected *(3 versions of product presented in packages, only)*, and informed scenarios *(3 versions of product presented in cups for participants to taste; packages with corresponding FoP labels also presented)* | Grape nectar Chocolate milk  3 versions of each product created to represent 1) control sugar condition (corresponding to added sugar in the marketplace), 2) a slightly sugar-reduced version, and 3) a highly sugar-reduced version | 1. Traffic light label 2. Nutrient text warning label: Black octagon with text “high in sugar” | **Behavior: P**articipants had to select which of the 3 products they wanted to consume (the regular-sugar, the slightly reduced sugar version, or the highly reduced sugar version). |
| **Lima et al., 2019 [17]** | School | Brazil  Children age 6-12y; n=492; 48% female  Education: Public school (54%); private school (46%)  Recruited from: 4 schools (2 public, 2 private) in Rio de Janeiro and Rio Pomba. | **Between-person:** Participants were randomized to one of 3 labeling conditions  **Control:** None (packages in all conditions carried FoP label) | 6 packaged foods (chocolate milk, sandwich cookies, frosted corn flakes, gelatin, yogurt, and corn snack)  3 unpackaged foods (Ice cream, banana, broccoli) | 1. Nutrient text warning: black octagons with “high in” sugar, saturate fat, sodium, or calories 2. Traffic light label 3. Guideline Daily Amounts | **Affect:** Children rated how they would feel eating the product by selecting all the emojis with the corresponding expression (including 16 emojis ranging from smiling, to neutral, to confused or sad) |
| **Machín et al., 2019 [18]** | Laboratory | Uruguay  Adults age ≥18y; n=199; 66% female  Education: 65% had a person with a university degree in household  Recruited from: Convenience sample of bread-consuming students and workers from the Universidad de la República and workers from the Ministry of Social Development. | **Between-person:** Participants were randomized to one of 2 labeling conditions  **Control**: No FoP label | Packaged bread  15 products from 6 categories (including cereal bars, crackers, cookies, alfajores (typical Uruguayan cookie-like sweets), unpackaged fruit, and peanuts) | 1. Nutrient text warning: black octagons with “Excess” sugar, saturated fat, and sodium 2. Control: no FoP label | **Attention**: Fixations on nutritional warnings  **Behavior:** Selection of a snack |
| **Egnell et al, 2019 [19]** | Online | The Netherlands  Adults age ≥18y; n=1,032; 49.9% female  Education: 1.3% primary, 30.4% secondary, 26.8% trade certificate, 31.9% university, 9.6% post-graduate  Recruited from: Convenience sample recruited from a web panel provider (PureProfile). | **Between-person:** Participants were randomized to one of 5 labeling conditions  **Control:** No FoP label **(w**ithin-person) | Packaged foods: pizzas, cakes, and breakfast cereals.  3 products within each category, ranging from least  to most healthy. | 1. Traffic lights: energy, fat, saturated fat, sugar, and salt 2. Reference intake: energy, fat, saturated fat, sugar, and salt 3. Nutrient text-warning: Black octagon with “high in” calories, sodium, saturated fat, and sugar, depending on the level 4. Nutri-score 5. Health Star Rating system | **Comprehension:** Participants ranked the set of 3 products according to their nutritional quality  **Message acceptance:** Liking, awareness, perceived cognitive workload, which were combined into dimensions through principal components analysis.  **Behavioral intentions: P**articipants selected which product they would be most likely to purchase. |
| **Talati et al, 2019 [20]** | Online | Argentina, Australia, Bulgaria, Canada, Denmark, France, Germany, Mexico, Singapore, Spain, the UK, and the USA  Adults age ≥18y; n=12,015; 50% female  Education: Not reported  Recruited from: International web panel provider. | **Between-person:** Participants were randomized to one of 5 labeling conditions  **Control:** No FoP label (within-person) |  | 1. Traffic lights: energy, fat, saturated fat, sugar, and salt 2. Reference intake: energy, fat, saturated fat, sugar, and salt 3. Nutrient text-warning: Black octagon with “high in” calories, sodium, saturated fat, and sugar, 4. Nutri-score 5. Health Star Rating system (energy, sat fat, sugars, sodium) | **Attention:** Participants rated whether label does not stand out.  **Comprehension:** Participants rated whether label: is easy to understand, takes too long to understand, is confusing, and provides the information they need.  **Message acceptance:** Participants rated how much they liked the label, trusted the label, and whether it should be compulsory for label to be shown on packaged foods. |
| **Ares et al, 2018 [21]** | Online | Uruguay  Adults age ≥18y; n=892; 66% female  Education: Primary (6%), secondary (67%), technical (12%), university (11%), post-graduate (3%)  Recruited from: Facebook advertisement targeting Facebook years >18y.  *Note: Only study 2 included.* | Between-person: Participants randomized to one of four labeling conditions  **Control:** No FoP label | Packaged lentils, green beans, breakfast cereal, yogurt, orange juice, bread, mayonnaise, and potato chips | 1. Nutrient text-warning: Black octagon with “high in” sodium, saturated fat, fat, and sugar, 2. Nutri-score 3. Health Star Rating system 4. Control: no FoP | **Comprehension:** Participants rated healthfulness of product.  **Behavioral intentions: P**articipants selected which product they would be most likely to purchase. |
| **Egnell et al, 2019 [22]** | Online | Germany  Adults age ≥18y; n=1,000; 50% female  Education: 10% primary, 38% secondary, 24% trade certificate, 13% university, 15% post-graduate  Recruited from: International web panel provider (Pure-Profile) | Between-person: Participants randomized to one of 5 labeling conditions  Control: No FoP label (within-person) | Packaged pizzas, cakes, and breakfast cereals | 1. Nutrient text warning: black octagon with “high in” sodium, saturated fat, and sugar 2. Nutri-score 3. Reference intakes: energy, sugars, fat, saturated fat, salt 4. Traffic lights: energy, sugar, fat, saturated fat, salt 5. Health star rating: energy, saturated fat, sugar, sodium | **Comprehension: P**articipants ranked set of 3 products according to nutritional quality |

1. Bollard, T.; Maubach, N.; Walker, N.; Ni Mhurchu, C. Effects of plain packaging, warning labels, and taxes on young people's predicted sugar-sweetened beverage preferences: an experimental study. *Int J Behav Nutr Phy* **2016**, *13*, 1-7, doi:10.1186/s12966-016-0421-7.

2. Arrúa, A.; Curutchet, M.R.; Rey, N.; Barreto, P.; Golovchenko, N.; Sellanes, A.; Velazco, G.; Winokur, M.; Giménez, A.; Ares, G. Impact of front-of-pack nutrition information and label design on children's choice of two snack foods: Comparison of warnings and the traffic-light system. *Appetite* **2017**, *116*, 139-146, doi:10.1016/j.appet.2017.04.012.

3. Neal, B.; Crino, M.; Dunford, E.; Gao, A.; Greenland, R.; Li, N.; Ngai, J.; Mhurchu, C.N.; Pettigrew, S.; Sacks, G., et al. Effects of different types of front-of-pack labelling information on the healthiness of food purchases—a randomised controlled trial. *Nutrients* **2017**, *9*, doi:10.3390/nu9121284.

4. Acton, R.B.; Hammond, D. Do consumers think front-of-package "high in" warnings are harsh or reduce their control? A test of food industry concerns. *Obesity* **2018**, *26*, 1687-1691, doi:10.1002/oby.22311.

5. Acton, R.B.; Hammond, D. The impact of price and nutrition labelling on sugary drink purchases: Results from an experimental marketplace study. *Appetite* **2018**, *121*, 129-137, doi:10.1016/j.appet.2017.11.089.

6. Egnell, M.; Talati, Z.; Hercberg, S.; Pettigrew, S.; Julia, C. Objective understanding of front-of-package nutrition labels: An international comparative experimental study across 12 countries. *Nutrients* **2018**, *10*, doi:10.3390/nu10101542.

7. Goodman, S.; Vanderlee, L.; Acton, R.; Mahamad, S.; Hammond, D. The impact of front-of-package label design on consumer understanding of nutrient amounts. *Nutrients* **2018**, *10*, doi:10.3390/nu10111624.

8. Khandpur, N.; de Morais Sato, P.; Mais, L.A.; Bortoletto Martins, A.P.; Spinillo, C.G.; Garcia, M.T.; Urquizar Rojas, C.F.; Jaime, P.C. Are front-of-package warning labels more effective at communicating nutrition information than traffic-light labels? A randomized controlled experiment in a Brazilian sample. *Nutrients* **2018**, *10*, doi:10.3390/nu10060688.

9. Lima, M.; Ares, G.; Deliza, R. How do front of pack nutrition labels affect healthfulness perception of foods targeted at children? Insights from Brazilian children and parents. *Food Qual Prefer* **2018**, *64*, 111-119, doi:10.1016/j.foodqual.2017.10.003.

10. Machín, L.; Arrúa, A.; Giménez, A.; Curutchet, M.R.; Martínez, J.; Ares, G. Can nutritional information modify purchase of ultra-processed products? Results from a simulated online shopping experiment. *Public Health Nutr* **2018**, *21*, 49-57, doi:10.1017/S1368980017001185.

11. Machín, L.; Aschemann-Witzel, J.; Curutchet, M.R.; Giménez, A.; Ares, G. Does front-of-pack nutrition information improve consumer ability to make healthful choices? Performance of warnings and the traffic light system in a simulated shopping experiment. *Appetite* **2018**, *121*, 55-62, doi:10.1016/j.appet.2017.10.037.

12. Acton, R.B.; Jones, A.C.; Kirkpatrick, S.I.; Roberto, C.A.; Hammond, D. Taxes and front-of-package labels improve the healthiness of beverage and snack purchases: a randomized experimental marketplace. *Int J Behav Nutr Phy* **2019**, *16*, N.PAG-N.PAG, doi:10.1186/s12966-019-0799-0.

13. Ang, F.J.L.; Agrawal, S.; Finkelstein, E.A. Pilot randomized controlled trial testing the influence of front-of-pack sugar warning labels on food demand. *BMC Public Health* **2019**, *19*, 1-8, doi:10.1186/s12889-019-6496-8.

14. Grummon, A.H.; Hall, M.G.; Taillie, L.S.; Brewer, N.T. How should sugar-sweetened beverage health warnings be designed? A randomized experiment. *Prev Med* **2019**, *121*, 158-166, doi:10.1016/j.ypmed.2019.02.010.

15. Khandpur, N.; Mais, L.A.; Sato, P.D.; Martins, A.P.B.; Spinillo, C.G.; Rojas, C.F.U.; Garcia, M.T.; Jaime, P.C. Choosing a front-of-package warning label for Brazil: A randomized, controlled comparison of three different label designs. *Food Res Int* **2019**, *121*, 854-861, doi:10.1016/j.foodres.2019.01.008.

16. Lima, M.; de Alcantara, M.; Ares, G.; Deliza, R. It is not all about information! Sensory experience overrides the impact of nutrition information on consumers’ choice of sugar-reduced drinks. *Food Qual Prefer* **2019**, *74*, 1-9, doi:10.1016/j.foodqual.2018.12.013.

17. Lima, M.; de Alcantara, M.; Martins, I.B.A.; Ares, G.; Deliza, R. Can front-of-pack nutrition labeling influence children's emotional associations with unhealthy food products? An experiment using emoji. *Food Res Int* **2019**, *120*, 217-225, doi:10.1016/j.foodres.2019.02.027.

18. Machín, L.; Curutchet, M.R.; Giménez, A.; Aschemann-Witzel, J.; Ares, G. Do nutritional warnings do their work? Results from a choice experiment involving snack products. *Food Qual Prefer* **2019**, *77*, 159-165, doi:10.1016/j.foodqual.2019.05.012.

19. Egnell, M.; Talati, Z.; Gombaud, M.; Galan, P.; Hercberg, S.; Pettigrew, S.; Julia, C. Consumers’ responses to front-of-pack nutrition labelling: Results from a sample from the Netherlands. *Nutrients* **2019**, *11*, 1817.

20. Talati, Z.; Egnell, M.; Hercberg, S.; Julia, C.; Pettigrew, S. Consumers’ perceptions of five front-of-package nutrition labels: An experimental study across 12 countries. *Nutrients* **2019**, *11*, 1934.

21. Ares, G.; Varela, F.; Machín, L.; Antúnez, L.; Giménez, A.; Curutchet, M.R.; Aschemann-Witzel, J. Comparative performance of three interpretative front-of-pack nutrition labelling schemes: Insights for policy making. *Food Qual Prefer* **2018**, *68*, 215-225.

22. Egnell, M.; Talati, Z.; Pettigrew, S.; Galan, P.; Hercberg, S.; Julia, C. Comparison of front-of-pack labels to help German consumers understand the nutritional quality of food products. Color-coded labels outperform all other systems. *Ernahrungs Umschau* **2019**, *66*, 76-84.

|  |  |
| --- | --- |
